# Cultivation type, season, and soil nematode interactions affect wheat rhizosphere metabarcoding profiles

**DOI:** 10.3389/fpls.2026.1869384

**Published:** 2026-07-16

**Authors:** Mariantonietta Colagiero, Isabella Pentimone, Aurelio Ciancio, Pasqua Veronico, Francesca De Luca, Elena Fanelli, Carlo Porfido, Claudio Cocozza, Laura Cristina Rosso

**Affiliations:** 1 Consiglio Nazionale delle Ricerche, Istituto per la Protezione Sostenibile delle Piante, Bari, Italy; 2 Department of Soil, Plant and Food Science, University of Bari “Aldo Moro”, Bari, Italy

**Keywords:** 16S rRNA gene, ASV, microbiota, nematodes, *Triticum*

## Abstract

**Introduction:**

The aim of this study was the evaluation of bacterial profiles and diversity in the rhizosphere of durum wheat, *Triticum turgidum* subsp. *durum*, in two different crop management types (organic vs. conventional) and at two sampling times. We investigated the links of rhizosphere bacteria with crop management and other variables, including soil nematodes and physicochemical profiles.

**Methods:**

Metabarcoding samplings were carried out in organic and conventional durum wheat fields at Gravina (Italy), before (March) and after (May) heading, including adjacent uncultivated controls. Bacterial amplicon sequence variants (ASVs) were obtained by RNA extraction from rhizosphere soil, followed by NGS sequencing of the V3–V4 regions of the 16S rRNA ribosomal gene. The nematodes were classified as herbivores, fungivores/moss feeders, bacterivores, and omnivores/predatory.

**Results:**

Crop fertilization and sampling time were effective drivers interacting with soil physicochemical traits and nematode guilds. Spearman’s correlation showed, in March, an inverse relationship of herbivores with all other guilds. In May, the herbivores were inversely correlated with the fungal/moss feeders. The sampling season affected the correlations among nematode guilds as well as ASV profiles, in both the control and cultivated samples. ASV diversity indexes did not vary significantly among treatments, except for the Menhinick index in the organic wheat control. In general, a shift toward a richer ASV metabarcoding profile was observed in May for both crop types, with a higher diversity and abundance in the organic wheat. Crop management type, sampling time, and soil physicochemical properties significantly affected the rhizosphere microbiota, with Alphaproteobacteria (Methylobacteriaceae and Sphingomonadaceae) as the most represented in both wheat crops. Positive or inverse correlations linked nematode groups and ASV, with profiles almost specific for each guild. Bacteria inversely correlated with herbivores included *Bacillus* sp., *Methylorubrum* sp., uncl. Lactobacillales, Cyanobacteria, and other taxa. Positive correlations included plant growth promoters and bacteria involved in N_2_ and nutrient cycling.

**Conclusions:**

The ASV profiles differed by management type and sampling time, as the sampling season affected the bacterial community, with the organic wheat always characterized by a higher richness and service redundancy. The occurrence of bacteria selectively associated with each nematode guild suggests the presence of multiple functional services deployed at the nematode–bacteria–rhizosphere interface.

## Introduction

Services such as functional diversity, plant nutrition, and pest regulation contribute to the long-term stability and resilience of soil ecosystems and microbial communities (Brussaard, 2013**;** Baer and Birgé, 2018**;** Xun et al., 2024). Most crop practices exert direct or indirect effects on soil microorganisms and, ultimately, on plant productivity. In particular, the microbial communities associated with plants include beneficial rhizosphere species such as plant growth-promoting rhizobacteria (PGPR), biocontrol agents, organic matter decomposers, and nutrient mobilizers (Schippers et al., 1987**;** Kokalis-Burelle et al., 2006**;** Anderson and Habiger, 2012**;** Giannelli et al., 2024**;** Xun et al., 2024). Field experimental data highlighted their involvement in the productivity of crops fundamental for food production and security such as cereals and wheat, *Triticum* spp. (Anderson and Habiger, 2012**;** Curtis and Halford, 2014**;** Grote et al., 2014**;** Kavamura et al., 2021**;** Chen et al., 2022**;** Fouad et al., 2025).

Wheat grains represent a top food commodity, and issues affecting their production and trade become special economic, political, and social concerns (Enghiad et al., 2017**;** Erenstein et al., 2022). Wheat genotypes have been the object, for many years, of intense genetic pressure and selection. However, in the last century, the broad genetic diversity of wheat progressively decreased due to the selection and adoption of a few cultivars with enhanced intensification and productive traits. Moreover, most modern genotypes highly depend on farming inputs such as chemical fertilizers and become more susceptible to biotic and abiotic stress factors (Acuña et al., 2023**;** Azarbad et al., 2022**;** Kinnunen-Grubb et al., 2020). The actual levels of wheat intensification, coupled with market and policy-related demands for safe practices and circular economy goals, require the identification and exploitation of sustainable tools, including beneficial microbial taxa, to maintain crop health and yields (Anikwe and Ife, 2023). For this purpose, more information is needed at the regional and field scales, as the mechanisms underlying wheat microbiota profiles, and related services, are locally variable, depending on factors such as plant genotype, growth stage and compartment, climate, and time of cropping (Schiro et al., 2019**;** Azarbad et al., 2022; Quiza et al., 2023**;** Schmidt et al., 2024).

Recent studies focusing on the microbiota of the wheat rhizosphere, a highly metabolically active and dynamic plant microenvironment, highlighted the effect of domestication and selection on microbial diversity (Kinnunen-Grubb et al., 2020**;** Reid et al., 2024). Reports include PGPR and beneficial species underpinning plant resistance or tolerance toward various stress sources, including drought and salinity (Timmusk et al., 2014**;** Schmidt et al., 2024**;** Fouad et al., 2025). Wheat genotype and related phenotypic plasticity are crucial factors affecting the rhizosphere microbiota, together with chemical inputs (Jacquiod et al., 2022**;** Quiza et al., 2023). The wheat rhizosphere microbiota also depends on seed-associated bacteria, owing to a sequence of microbial competition and cooperation processes involving early root colonization, trophic niche partitioning, and facilitation processes (Garrido-Sanz and Keel, 2025). Seed bacteria that first colonize the root at germination appear favored during this process and better outcompete with other soil bacteria. The metabolism of taxa specialized on root exudates eventually facilitates their enrichment in the community, owing to specialized microorganisms feeding on root exudates. A defined series of events hence favors the dominance, within the rhizophere microbiota, of seed-born vs. soil-born bacteria, highlighting a potential of beneficial taxa colonizing seeds (Garrido-Sanz and Keel, 2025).

Factors external to the seed–soil interaction that affect the wheat rhizosphere microbiota include, apart from plant genotype, crop management practices, soil type, and pests. Wheat-associated PGPR profiles showed a dependence on the rhizosphere/rhizoplane compartment and on anthropic factors such as plant fertilization and domestication (i.e., diploid vs. allopolyploid genotypes). In particular, chemical fertilizers showed a major impact on the abundance of PGPR, but mostly in modern wheat genotypes (Reid et al., 2024).

Given the diversity of rural environments and wheat intensification levels, any evaluation of the interactions of crop practices and rhizosphere microbiota requires farm-scale investigations. For this purpose, basic approaches include field surveys and monitoring, to identify mechanisms affecting microorganisms involved in plant nutrition, pest regulation, and conservation of soil functional diversity. The assessment of these services, leading to the protection or enhancement of best practices, should also consider crop management type (i.e., organic vs. conventional or locally traditional systems) due to the different inputs they rely on, including the use, in conventional cropping, of synthetic fertilizers, pesticides, and/or herbicides.

The beneficial effects of soil microbial communities on plant productivity, and their eventual adaptation to stress, also require sampling at different time scales, as well as increasing resolution by analyzing whole microbial communities through advanced, DNA-based techniques (Sudermann et al., 2024). This approach is crucial when investigating the distribution, diversity, and effects of microbial communities in relation to environmental variables (Sudermann et al., 2024). Metabarcoding has been successfully applied, in various crops, to determine and evaluate the links between symbionts and roots, to study root biology, or to identify the effect of limiting factors such as pathogens or pests (Reinhold-Hurek et al., 2015**;** Piombo et al., 2021**;** Kawasaki et al., 2021**;** Djotan et al., 2023**;** Giannelli et al., 2024). This approach has also been helpful to test the impact of pesticides, fertilizers, or plant management, in field or semi-controlled conditions (Xue et al., 2015**;** Kavamura et al., 2021).

Nematodes show several interactions with the surrounding bacteria in soil and are involved in crucial ecosystem processes. In particular, the rhizosphere microbiota includes biocontrol agents with different specialization levels, characterized by mechanisms of action ranging from direct nematode parasitism to antibiosis, exerted through metabolic by-products. Nematodes are involved in many processes, including regulation or dispersal of soil bacteria, pests, or pathogens, as well as organic matter decomposition and nutrient mobilization. Consequently, conservation efforts should aim at preserving natural resources such as soil microorganisms and invertebrates, as they are directly or indirectly involved in crop production.

We herein report the results of a metabarcoding study evaluating bacterial profiles and diversity in the rhizosphere of durum wheat, *Triticum turgidum* subsp. *durum* (Desf.) Husn., under two different crop types. The study was carried out, at two sampling times, in two wheat fields with either an organic or a conventional management, planted with the same cv. Adjacent uncultivated sites were included as background controls. Our aim was to determine whether changes occur in rhizosphere bacteria communities by comparing both fields and the practices therein applied, eventually assessing the correlation between rhizosphere taxa with crop management and other factors, including soil nematode communities and chemical profiles.

## Materials and methods

### Samplings

Rhizosphere soil samples were collected from plants of the mid-late, modern durum wheat *cv* Beltorax (AABB, resistant to rust and *Septoria tritici* blotch, with a medium-early heading time), cultivated in two fields at Gravina (Italy). The fields were selected due to the same genotype and seeds therein used, to exclude differences related to the seed microbiota. The first field (40° 52’ 50.5’’ N, 16° 20’ 13.3’’ E) was managed following an organic crop protocol (Regulation EU 2018/848) for at least 5 years, including an annual rotation with *Vicia sativa* L. Foliar fertilization was performed between the end of the tillering stage and the beginning of the stem extension stage using a commercial fertilizer, allowed in organic agriculture, containing hydrolyzed protein, and microelements (B, Mn and Zn; Ambition^®^ Aktivator, Bayer, Italy). The second field (40° 49’ 48.0’’ N, 16° 26’ 43.3’’ E) followed a local, traditional management herein indicated as “conventional”, including a bi-annual rotation with *V. sativa*, soil fertilization with NH_4_NO_3_, weed control through the application of synthetic herbicides, and the use of fungicides. The multi-annual average yields were 2.5 and 4.0 ton/ha for the organic and conventional wheat, respectively. The fields were sampled twice in the year 2023, before flowering (March) and after heading (May), collecting five replicated 2-L samples of soil and roots, at an average depth of 15–20 cm, from plants located at approximately a 10-m interval along a North–South diagonal field transient. Five replicated background control samples were also collected for each field and sampling time from adjacent wheat-free sites, placed at 10–20 m from the sampled crops, at the field entrance and in uncultured edge areas, collecting soil and weed roots as for the wheat plants. The sampled weeds included mixed assemblages of spontaneous species such as *Malva sylvestris* L., *V. sativa* L., *Trifolium repens* L., *Cirsium arvense* L. (Scop.), *Echium plantagineum* L., *Rumex obtusifolius* L., *Galium aparine* L., and other members of Apiaceae and Poaceae. A total of 40 samples were collected for this study. After collection, subsamples (approximately 300 g of mixed soil and roots) were stored at −80°C, for collection of rhizosphere soil and subsequent processing for metabarcoding analyses. The remaining soil was used for nematode or soil analyses.

The samples were classified by variables such as cultivation method, description (crop or control), fertilization applied (or not), type of plant (wheat/weed), time of sampling (March/May), number of nematode taxa, and levels of herbivores, bacterivores, fungal/moss feeders, and omnivorous-predatory nematodes, classified as density (nematodes/100 mL soil) and prevalence (percentage within total nematodes). The densities were classified, based on the sample observed values, as L = low density (≤25% of the total mean), M = medium density (within 25%–75% of mean), and H = high density (≥75% of mean). The samples were classified, for guild prevalence, as L = low (≤20% of the mean), M = medium (20%–50%), and H = high (≥50%).

The nematodes were extracted from each soil sample using the sieving and decanting technique, suspending a 200-mL soil subsample in tap water followed by filtering through a set of 500- and 75-µm sieves, with subsequent decanting. The filtered suspension was then examined for nematode identification and density counting with light microscopy, using a Hawksley counting chamber at 40×. Herbivores (*Helicotylenchus* sp. and other tylenchids) were identified at the species/genus level using available taxonomic data with a Leitz-Orthoplan light microscope, at 312–500×. The bacterivores (Rhabditida and Cephalobidae), the fungal/moss feeders (i.e., *Tylenchus*, *Filenchus*, and *Aphelenchoides* spp.), and the omnivore-predatory nematodes (Mononchida and non-plant parasitic Dorylaimida) were classified during counting, using a Hawksley counting chamber at 40–100× or temporary water mount slides, and available taxonomic descriptions.

The samples collected in March were air-dried and passed through a 2-mm sieve, and analyzed to determine physicochemical variables such as soil texture, pH, electric conductivity (EC), and C, N, P, and exchangeable cation content.

The particle size distribution was determined using a grain size analyzer (Gibertini, Italy) in accordance with the ISO 17892-4:2016^1^. The textural class of each soil was determined using the USDA soil texture calculator (https://www.nrcs.usda.gov/resources/education-and-teaching-materials/soil-texture-calculator).

The total inorganic carbon (TIC), total organic carbon (OC), and total nitrogen content of the soils were determined through automated combustion analysis (Elementar soli TOC cube, Germany) according to the ISO 10694:1995^2^ standard.

The remaining soil parameters were determined in accordance with the analytical methods of Sparks et al. (1996). In detail, the pH_KCl_ was measured suspending 10 g of each soil in 25 mL of 1 M KCl solution, while EC was measured in filtrates with a soil-to-water ratio of 1:2.

The available P (AP) was extracted with a 0.5 M NaHCO_3_ solution and determined spectrophotometrically at 650 nm.

The exchangeable bases (K, Mg, Ca, and Na) were extracted with a 10% (w/v) BaCl_2_ solution containing 22.5 mL of L-1 triethanolamine buffered at pH 8.2, and determined using an inductively coupled plasma optical emission spectrometry (iCAP 6000, Thermo Electron, USA).

### Metabarcoding analyses

A total of 40 rhizosphere soil samples were analyzed for bacterial profiles through a metabarcoding sequencing approach. For each sample and sampling time, 2 g of soil was collected from roots of wheat or control weeds, using a steel spatula washed and sterilized in 95% ethanol before each root processing. Total RNA was extracted with the RNeasy PowerSoil^®^ Total RNA Kit (Qiagen^®^, UK), following the manufacturer’s instructions. The RNA concentration was determined with a Nanodrop™ spectrometer at 260 nm. The extracted material was subjected to reverse transcription according to the sequencing protocol, using SuperScript IV (Invitrogen, USA). The material obtained was then purified using the PCR Purification Kit (Norgen, Biotek, UK). The nucleic acid integrity was checked by electrophoresis on 1.5% agarose gel.

The cDNA obtained was then subjected to PCR amplification of the bacterial V3–V4 hypervariable region in the 16S ribosomal RNA gene that was considered sufficient to yield information allowing taxonomic classifications (Yang et al., 2002; Liu et al., 2008; Caporaso et al., 2011). Libraries were prepared by following the Illumina 16S Metagenomic Sequencing Library Preparation protocol in two amplification steps: an initial 35-cycle PCR amplification using locus-specific PCR primers, and a subsequent amplification integrating relevant flow-cell binding domains and unique indexes (NexteraXT Index Kit, FC-131-1001/FC-131-1002). Both ends of the V3–V4 region were used for amplification using the primer sequences: 16S_341F (5′-CCTACGGGNGGCWGCAG-3’) and 16S_805R (5′-GACTACHVGGGTATCTAATCC-3’) (Van de Peer et al., 1996**;** Takahashi et al., 2014**;** Clarridge, 2004).

Libraries were sequenced by a commercial service (IGA-Technology, Services, Udine, Italy) on an AVITI instrument (Element Biosciences, San Diego, CA) using 300-bp paired-end mode. The raw data generated were deposited at National Center for Biotechnology Information (NCBI) Sequence Read Archive (BioProject acc. n. PRJNA1257288, available at https://www.ncbi.nlm.nih.gov/sra). The sequences were imported in the Galaxy online platform (https://usegalaxy.eu/ and https://usegalaxy.org/) (Jalili et al., 2020) and data were analyzed with QIIME2 (Bolyen et al., 2019). Reads were de-noised and merged through DADA2 pipeline analysis, based on amplicon sequence variants (ASVs) (Callahan et al., 2016). DADA2 produced a feature table containing high-resolution ASV whose taxonomy was assigned in the next step, based on the exact correspondence between ASV and reference strains present in the SILVA database (Quast et al., 2013). All retained taxonomies were finally checked, and eventually emended when needed, using the online DSMZ “Official List of Prokaryotic Names with Standing in Nomenclature” as a standard reference, available at https://lpsn.dsmz.de/ (accessed on 9 June 2025) (Göker et al., 2025). A few (<10) taxonomies were emended using the MIDAS database (Dueholm et al., 2024).

### Data analysis

The ASV.biom table and the related mapping file with samples description were used for further analyses with R ver. 0.1.1.2 (R Core Team, 2013). The library *mctoolsr* (Leff and Fierer, 2013) was used to produce the Bray–Curtis dissimilarity matrices and NMDS samples plots, using the whole dataset or sample groups, as well as for the Kruskal–Wallis *t*-test and PERMANOVA (permutational analysis of variance) analyses. Further R libraries included *ggplot2* (Wickham, 2016) for graphics. Correlations for ASV and/or nematode variables were calculated with R using library *psych* (Revelle, 2017), or using PAST (Hammer et al., 2001). PAST was also used to calculate and compare α-diversity indices of samples. Venn diagrams were produced online at http://bioinformatics.psb.ugent.be/webtools/Venn/ (Heberle et al., 2015).

Sample ASV data grouped by different variables were also analyzed using STAMP (Statistical Analysis of Metagenomic Profiles, ver. 2.1.3; http://kiwi.cs.dal.ca/Software/STAMP), applying an equal variance two-sided *t*-test (*p* ≤ 0.05) with a 95% confidence interval and a ratio of proportions ≤ 2 as effect size filter (Parks et al., 2014). Comparisons included sample groups identified by mapping variables, i.e., crops vs. controls, management type and sampling time, conventional vs. organic wheat, and density or percentage of herbivores or other nematode groups. The entire samples were used as parent level, applying a two-tailed Student’s *t*-test at different taxonomic levels, with other comparative statistics. The unclassified taxa, and higher levels, were identified in the analyses (and eventually represented in STAMP plots) by using the lowest ASV classification or code as a tag for higher hierarchies. Heatmaps or bar plots of significantly different ASV (ANOVA, with 0.95 *post-hoc* Tukey–Kramer test, filtering threshold: *p* ≤ 0.05) were shown with trees produced with average neighbor UPGMA and a 0.65 dendrogram clustering threshold.

## Results

### Soil physicochemical traits

The texture of both field samples was clay or clay loam with similar amounts of sand (means range = 26.5%–31.4%). The conventional wheat samples showed a significantly higher silt content when compared with the organic wheat and its adjacent control (37.18% vs. 29.9% and 29.3%, respectively, Student’s *t* test *p* ≤ 0.01 and < 0.05). The organic wheat soil and its control also showed a clay content significantly higher than the other conventional treatments (*p* ≤ 0.001 to 0.05, **Supplementary Table 1A**).

Principal component analysis (PCA) based on correlation matrix of all variables showed close sample coordinates and groups. The PCA plan allowed sample aggregation along the first and second axes in relation to the field (organic vs. conventional) and origin (cropping vs. uncultivated control, respectively). Both axes accounted for >60% of total variance (**Supplementary Figure 1**).

Soil pH values were close to neutral across all groups (range = 6.97–7.18), with conventional crop showing significantly higher values than organic wheat field (7.18 vs. 6.97, respectively, *p* < 0.05). No significant difference was found among samples for soil AP content, although this variable was higher in both conventional treatments. The conventional wheat samples also showed total inorganic C contents significantly higher than in the organic wheat (1.34% vs. 0.89%, respectively, *p* < 0.001). The conventional controls also showed, when compared with the organic controls, higher values of organic C (1.99% vs. 1.38%, *p* ≤ 0.01) and N (0.23 vs. 0.15%, *p* ≤ 0.05). The conventional wheat samples had lower C/N values compared to the organic wheat (8.8 vs. 9.9, *p* ≤ 0.05) (**Supplementary Tables 1B–D**). They were also characterized by a higher mineral content, as shown by the EC values, which were the highest and differed from all other groups (*p* ≤ 0.05). No difference was found between the organic wheat and its control for the exchangeable cation contents (Ca^++^, Mg^++^, K^+^, and Na^+^). The conventional wheat showed instead a Ca^++^ content significantly lower than the organic crop (*p* ≤ 0.01), whereas Mg^++^ and K^+^ levels were significantly higher (*p* ≤ 0.01 and 0.05, respectively). Compared to its control, the conventional wheat samples also showed higher Mg^++^ and lower K^+^ contents (*p* ≤ 0.05) (**Supplementary Tables 1E, F**).

### Nematodes

Nematode populations showed, at both sampling times, the presence of different herbivore taxa including *Helicotylenchus tunisiensis, Histotylenchus* sp.*, Paratylenchus sheri, Pratylenchus neglectus, Merlinius* sp., a criconematid, and, in one conventional control sample only, *Xiphinema pachtaicum* and *Heterodera* sp. The groups of fungivores and moss feeders included tylenchids such as *Tylenchus* sp., *Psilenchus* sp., *Aphelenchoides* sp., and small dorylaims, such as *Crassolabium* sp. Bacteria-feeding nematodes included Rhabditidae, Cephalobidae, *Acrobeles* sp., *Tobrilus* sp., a plectid, and other aquatic taxa. Omnivore and predatory nematodes included species of *Thonus*, *Discolaimus*, *Belondira*, *Ecumenicus monohystera*, a mononchid, and two other dorylaims, differentially represented among the samples.

**Figure 1:**
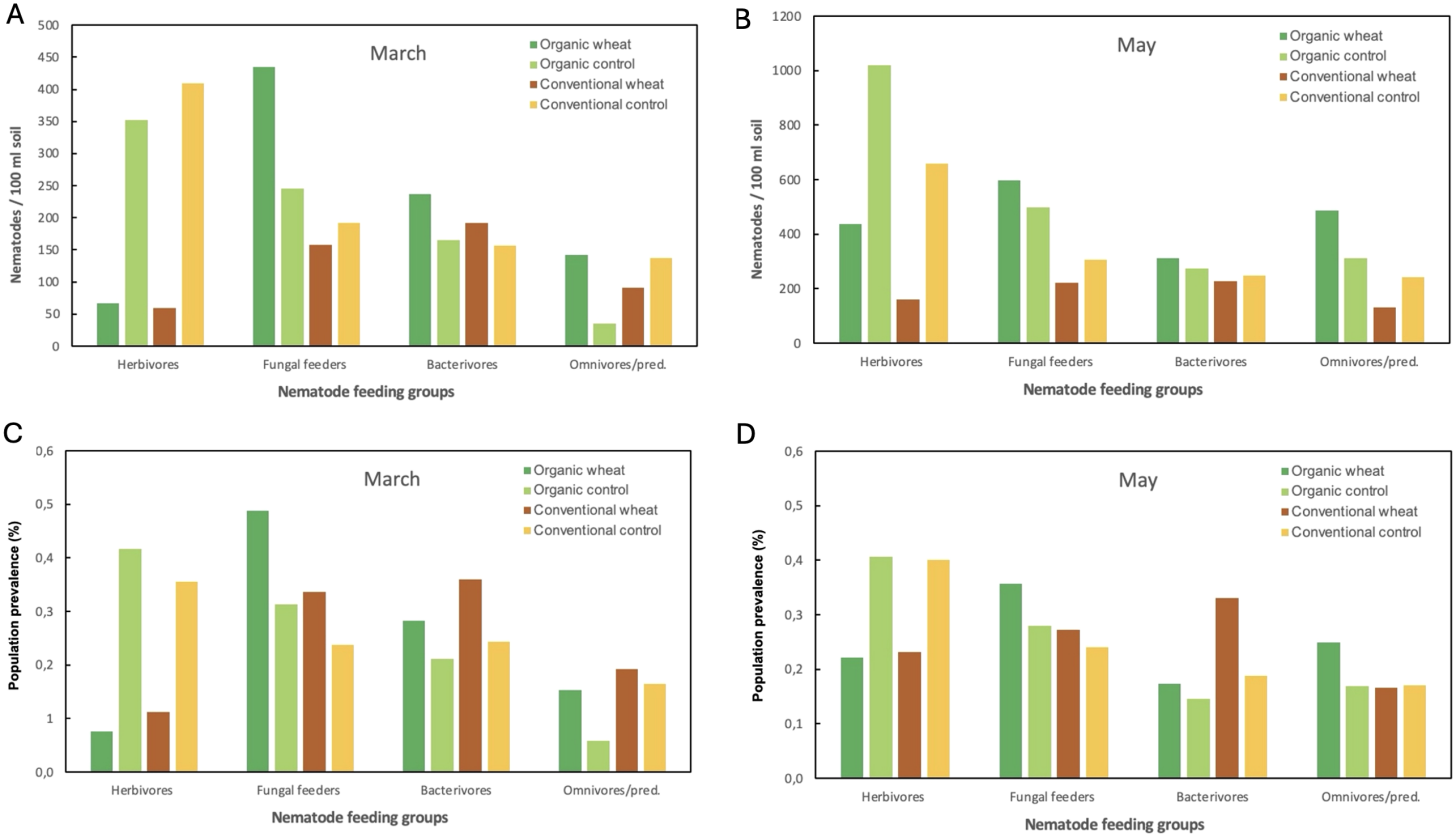
Density **(A, B)** and population prevalence **(C, D)** of rhizosphere nematodes classified in four feeding groups in the conventional and organic field samples and by sampling times, including background uncultivated controls.

The feeding groups showed consistent trends, when considering the sample types and seasons. The herbivores, considered as both densities and population prevalence, were always higher in the control samples than the corresponding wheat crops, irrespective of management types and sampling times. In contrast, fungal feeders were abundant in organic wheat, in both density and prevalence. Similarly, the bacterivores and omnivores/predators showed, at both sampling times, the highest densities in the organic wheat. However, the bacterivore prevalence peaked in the conventional wheat, at both sampling times (**Figure 1**).

The Spearman’s ranking correlations showed that nematode feeding groups occurred at the first sampling time with mutually exclusive trends. The herbivore densities were inversely correlated with the prevalence (%) of fungal/moss feeders, bacterivores (*p* ≤ 0.05), and omnivore/predatory species (*p* ≤ 0.01). This trend was also significant when considering the prevalence of herbivores in the populations. The samples collected in May confirmed an inverse relationship of herbivores, but only with the numbers of fungal/moss feeders (*p* < 0.05) and bacterivores (*p* < 0.01), and their population prevalence (*p* < 0.001 and *p* < 0.01, respectively). The herbivore densities in May, however, showed positive correlations with those of bacterivores (*p* < 0.05) and omnivores (*p* < 0.001), both also positively correlated (*p* < 0.01). At this sampling time, a general trend toward increased nematode multiplication rates was observed, as shown by the higher densities and the almost double mean number of taxa found per sample (6.4 in March vs. 12.6 in May). This variable reflected the appearance of rare or less represented species, correlated to the numbers of herbivores, bacterivores (*p* < 0.05), and omnivores/predators (*p* < 0.001), with the only exception of the fungal and moss feeders (**Table 1**).

**Table 1:** Spearman's ranking correlations (*p*) among nematodes feeding groups in the rhizosphere of conventional and organic wheat cv Belthorax, based on density and prevalence data collected in two fields at Gravina and at two sampling times (March and May).

March	Herbivores (density^1^)	Fungal / moss feeders (density)	Bacterivores (density)	Omnivores. predators (density)	N. of taxa	Herbivores (prevalence^2^)	Fungal / moss feeders (prevalence)	Bacterivores (prevalence)
Fungal / moss feeders (density)	0.0053							
Bacterivores (den.)	-0.0877	0.1418						
Omnivores. predators (den.)	-0.4192	0.1221	0.2583					
N. of taxa	0.1750	0.1212	0.0324	0.2573				
Herbivores (prevalence)	0.9711 ***	-0.1432	-0.1977	-0.4900	0.1006			
Fungi & moss feeders (prev.)	-0.4686 *	0.7209 ***	-0.1686	0.0329	-0.1099	-0.4866 *		
Bacterivores (prev.)	-0.4948 *	-0.4356	0.6852 **	0.2486	-0.0971	-0.4621 *	-0.2658	
Omnivores/ predators (prev.)	-0.6075 **	-0.1111	0.0474	0.8766 ***	0.1426	-0.6271 ***	-0.0011	0.3274
May								
Fungal / moss feeders (den.)	0.38299							
Bacterivores (den.)	0.5203 *	0.5206 *						
Omnivores / predators (den.)	0.7606 ***	0.6395 **	0.6392 **					
N. of taxa	0.5533 *	0.4068	0.5358 *	0.6876 ***				
Herbivores (prev.)	0.6674 *	-0.1949	-0.0843	0.1274	0.0670			
Fungal / moss feeders (prev.)	-0.5488 *	0.3183	-0.1849	-0.2021	-0.2613	-0.7035 ***		
Bacterivores (prev.)	-0.6548 **	-0.4168	0.0709	-0.4629 *	-0.2321	-0.5671 **	0.0945	
Omnivores / predators (prev.)	0.1875	0.2616	0.2264	0.6649 **	0.5599 *	-0.3306	0.1324	-0.0873

1 Density = number of nematodes / 100 ml soil, from five replicated samples for each treatment and sampling time (total observations = 40).

2 Prevalence = % within total nematode population.

3 In bold are reported the statistical significance (* = p<0.05; ** = p<0.01; *** = p<0.001).

### ASV diversity

A total of 38,038,540 sequences were produced by Qiime2 accounting for 2,363 ASVs, reduced to 37,826,130 after eliminating 718 singletons. To remove low-frequency artifacts, the 1,555 ASV table was further filtered applying a total % method (Drake et al., 2022), eliminating the entries with less than 0.01% of total dataset (using a threshold of 350 total sequences per ASV, as sum of all samples), yielding a final ASV table with 1,469 entries. A final dataset of 1,202 ASVs (including Bacteria and Archaea) was finally produced for subsequent metabarcoding analyses, filtering the taxa showing > 36 null values, to keep the ASV that appeared at least in one sampling time and treatment only (4/5 of replicates). The taxonomic profiles of these samples yielded a total of 47 phyla for Bacteria (including *Candidatus* phyla) and 3 for Archea (Methanobacteriota, Nanobdellota, and Thermoproteota). Including unclassified taxa, the ASV represented 109 classes (106 Bacteria and 3 Archea), 217 orders (4 Archea), 359 families (4 Archea), and 648 genera (4 Archea). The majority of ASVs were unclassified, with only 104 (8.6%) identified at the species level, whereas 373 were “uncultured” or “unclassified” and 34 showed a “*Candidatus*” status at the genus or higher taxonomic levels.

The diversity indexes, based on ASV sequence abundance at the species level, were calculated for all sample types and compared. Consistently with the similarity between fields, most indexes did not show significant variations by treatments and at both sampling times. However, a lower diversity could be ascribed to the conventional crop. The evenness and equitability indexes (range: 0.0–1.0), were lower in March for the conventional crop when compared with both controls (evenness value = 0.26, vs. 0.47 and 0.48 in the conventional and organic controls, respectively, *p* < 0.05). These indexes account for the distribution of individuals among the sample taxa, with lower levels in the conventional wheat, indicating a higher dominance of a few taxa. However, the significant differences found in March for the conventional crop were not observed again in May (**Supplementary Tables 2A, B**). In March, the conventional wheat crop also showed a higher Berger–Parker index (numerical dominance = proportion of most abundant taxon individuals on total, range: 0.0–1.0) than all other treatments, and significantly different from both controls (*p* < 0.05), consistent with a lower diversity present in the conventional wheat. In May, these indexes were still higher in conventional samples than in organic samples, but the differences were not significant. An effect was found in May only for the Menhinick richness index, which accounts for the richness of species, significantly higher in the organic field when comparing wheat crop vs. the organic control (**Supplementary Table 2B**). Menhinick richness index and Equitability_J showed a significant (*p* ≤ 0.05) decrease between the two sampling times, but only in the organic wheat controls, with lower means in May (**Supplementary Table 2C**).

### Metabarcoding profiles

**Figure 2:**
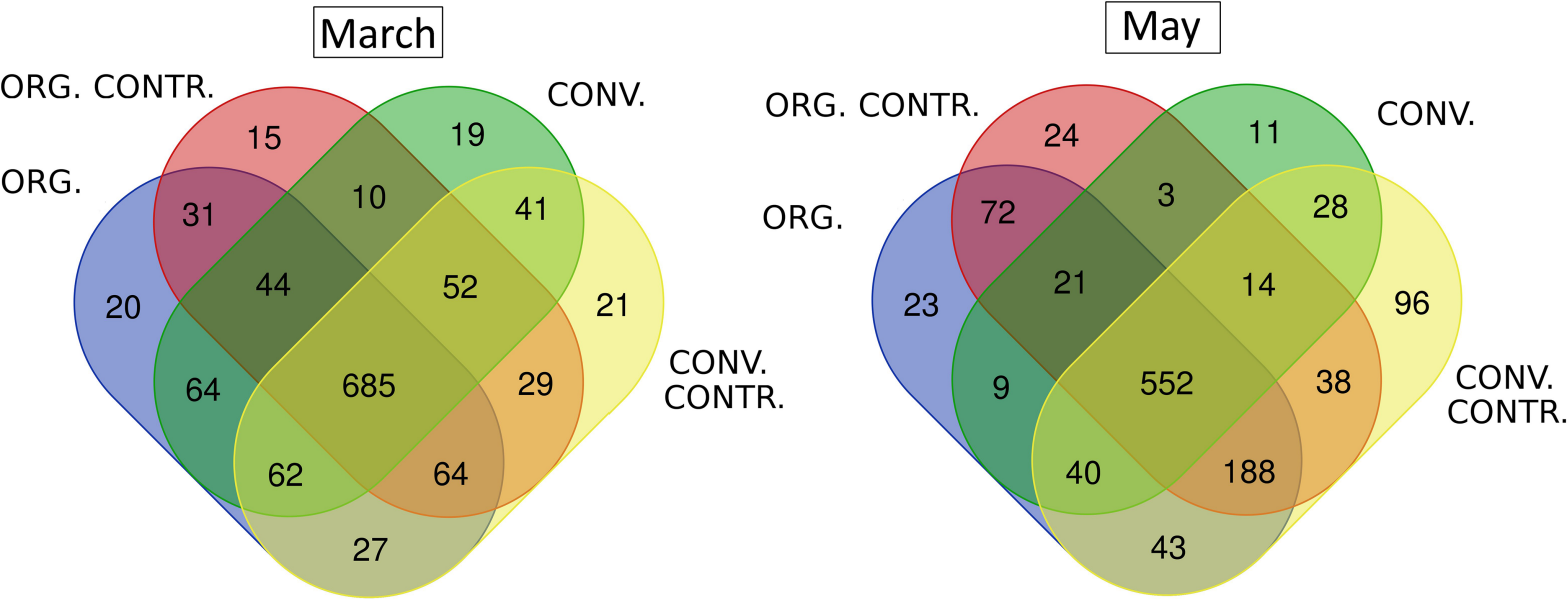
Venn diagrams showing ASV abundance by groups of samples and sampling times, in the conventional (“CONV.”) and organic (“ORG.”) wheat fields and background control samples. Values show the number of taxa present in five replicated samples per group, after dataset filtering (total ASV = 1,202).

Venn plots of organic, conventional wheat and related control samples showed a common core bacterial community accounting, in March and May, for 57.8% and 47.5% of ASV, respectively. Considering the core taxa, 492 ASVs were in common between the two sampling times, with 195 unique to March and 61 unique to May core groups. The ASV distributions showed a progressive decrease in May for all combinations that included the conventional wheat samples, whereas the taxa in the organic wheat and its control groups, and related combinations, increased (**Figure 2**). At both sampling times, each wheat group showed more than 800 core taxa in common with its control, except for the conventional field samples in May, which showed only 634 ASVs. A general reduction in taxa that were unique, per season and treatment, was observed in May, except for the conventional control that showed a 2.5-fold increase (**Supplementary Figure 2A**). Similarly, the effect of the sampling season showed a reduction in the taxa unique per group, between March and May, with a minor increase observed in May in the conventional control (**Supplementary Figure 2B**). When the data were pooled together and compared by sampling times, a large ASV fraction (96.9%) was found in common between the two samplings, with 23 unique ASVs appearing in March and 14 in May (**Supplementary Figure 2C**).

Partial linear correlations of samples based on ASV abundance (filtered at a total sum of 10,000 sequences per taxon) showed significant coefficients (*p* ≤ 0.05) both within the same sample groups and when comparing replications from different fields and groups, consistent with a similarity between the two fields for the most abundant taxonomic profiles and repartitions (**Supplementary Table 3**). Whittaker’s β-diversity indexes, calculated by sampling groups and times, showed low values confirming the high homogeneity of samples (**Table 2**). NMDS plots also confirmed homogeneity and consistently showed partial overlapping of sample clusters, for most variables examined (**Supplementary Figure 3**). PCA and cluster analysis based on ASV abundance, however, showed a separation of the organic from the conventional samples, more evident in May, with a tendency for conventional samples to form separate clusters (**Supplementary Figure 4**).

**Table 2:** Whittaker’s diversity indexes for samples groups and times^1^.

β diversity	March	May
Organic wheat	0.8825	0.5166
Organic controls	0.6373	0.4903
Conventional wheat	0.9517	0.7648
Conventional controls	0.6189	0.9786
Global (all samples)	1.1526	1.0423

^1^Calculated with PAST, total ASV dataset (1,202 ASV).

**Figure 3:**
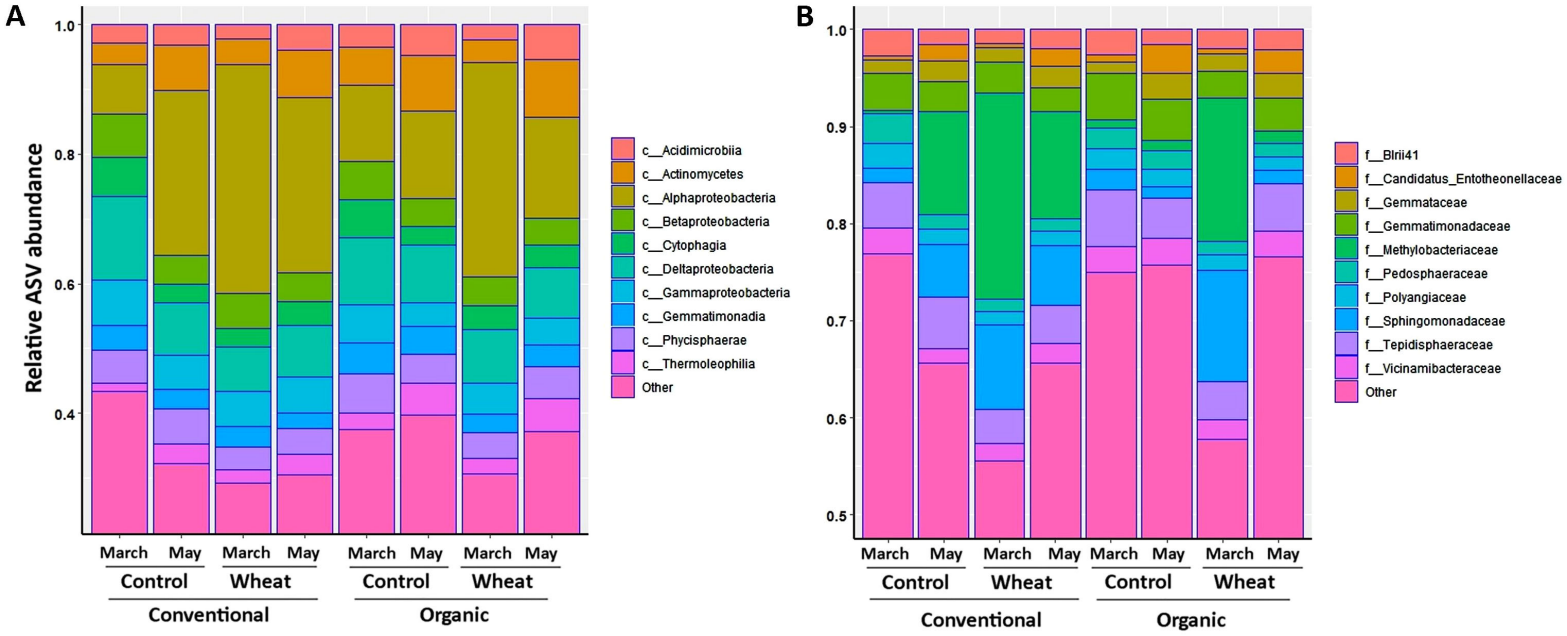
Relative abundance and distribution of dominant (top 10) ASV taxa at the class **(A)** and family **(B)** levels, found in the conventional and organic wheat fields and related controls, by sampling times.

The most abundant (top 10) bacterial classes per field and sampling time are shown in **Figure 3A**. The Alphaproteobacteria were the most represented in the wheat samples, showing abundance higher than the controls in March, and almost equivalent in May, in both organic and conventional fields. In May, the Alphaproteobacteria increased in the conventional controls and remained stationary in the organic controls, but decreased between the two sampling times in both wheat fields. At the family level, most differences of Alphaproteobacteria were observed in March for members of Methylobacteriaceae and Sphingomonadaceae, most represented in the conventional and organic wheat samples (**Figure 3B**).

Significant (*p* ≤ 0.05) PERMANOVA pairwise comparisons revealed discernible effects when comparing conventional with organic samples (including controls), and by sampling times, presence of fertilization, herbivore density, and the fungal/moss feeders (as percentage). No difference was observable when comparisons were based on soil variables. An effect was also evident when comparing the samples, classified as wheat or controls, for each sampling time (**Supplementary Table 4**).

**Figure 4:**
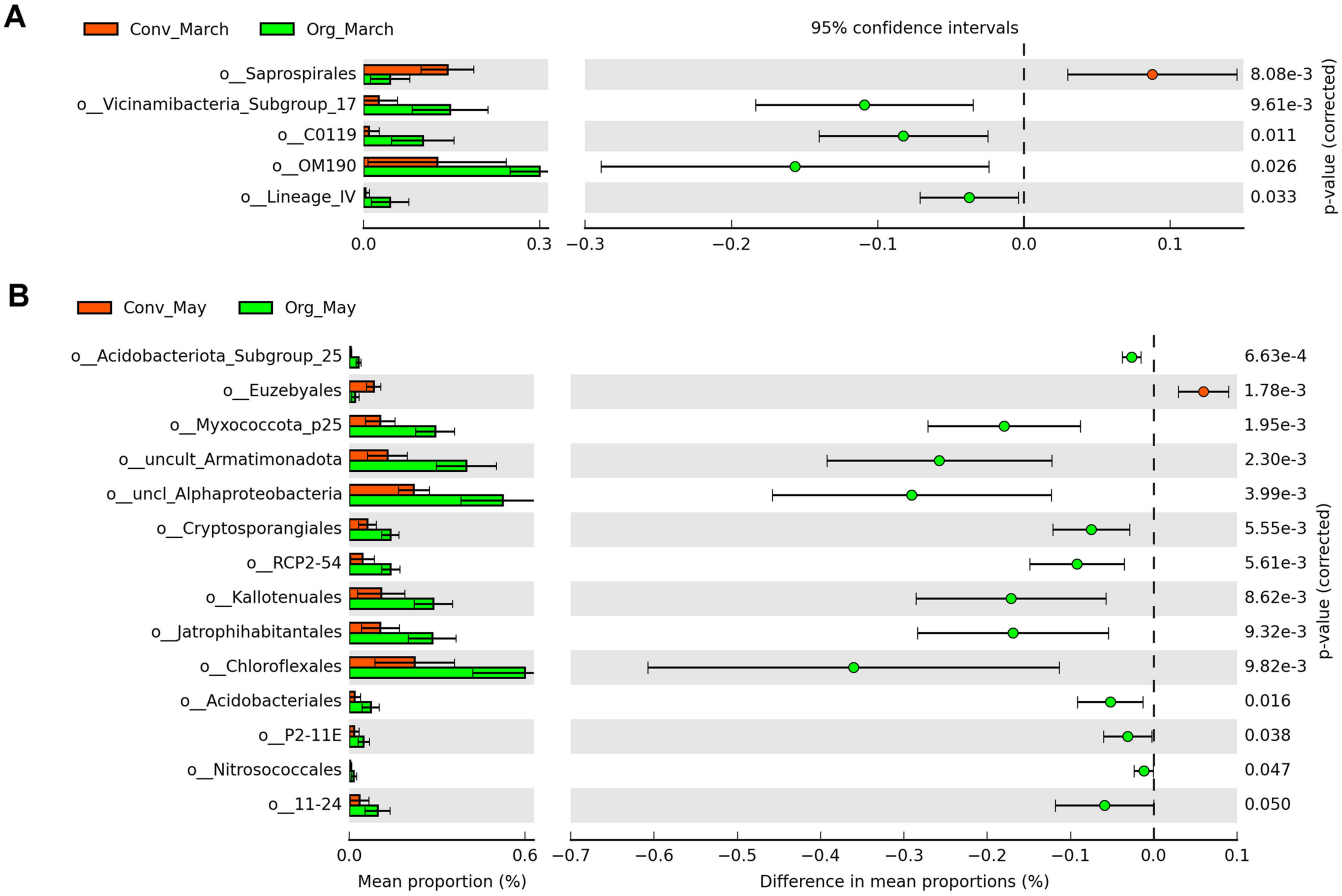
Differential ASV soil rhizosphere profiles between the conventional and organic wheat plants, shown at the order level in the March **(A)** and May **(B)** samplings.

Based on PERMANOVA results, we compared the ASVs differentially represented between the organic and the conventional managements. The more represented taxa in the organic field in March were members of orders OM190 (p_Planctomycetes), C0119 (p_Chloroflexi), and c_Vicinamibacteria (p_Acidobacteriota), with Saprospirales (p_Bacteroidota) more abundant in the conventional field (**Figures 4A, B**).

**Figure 5:**
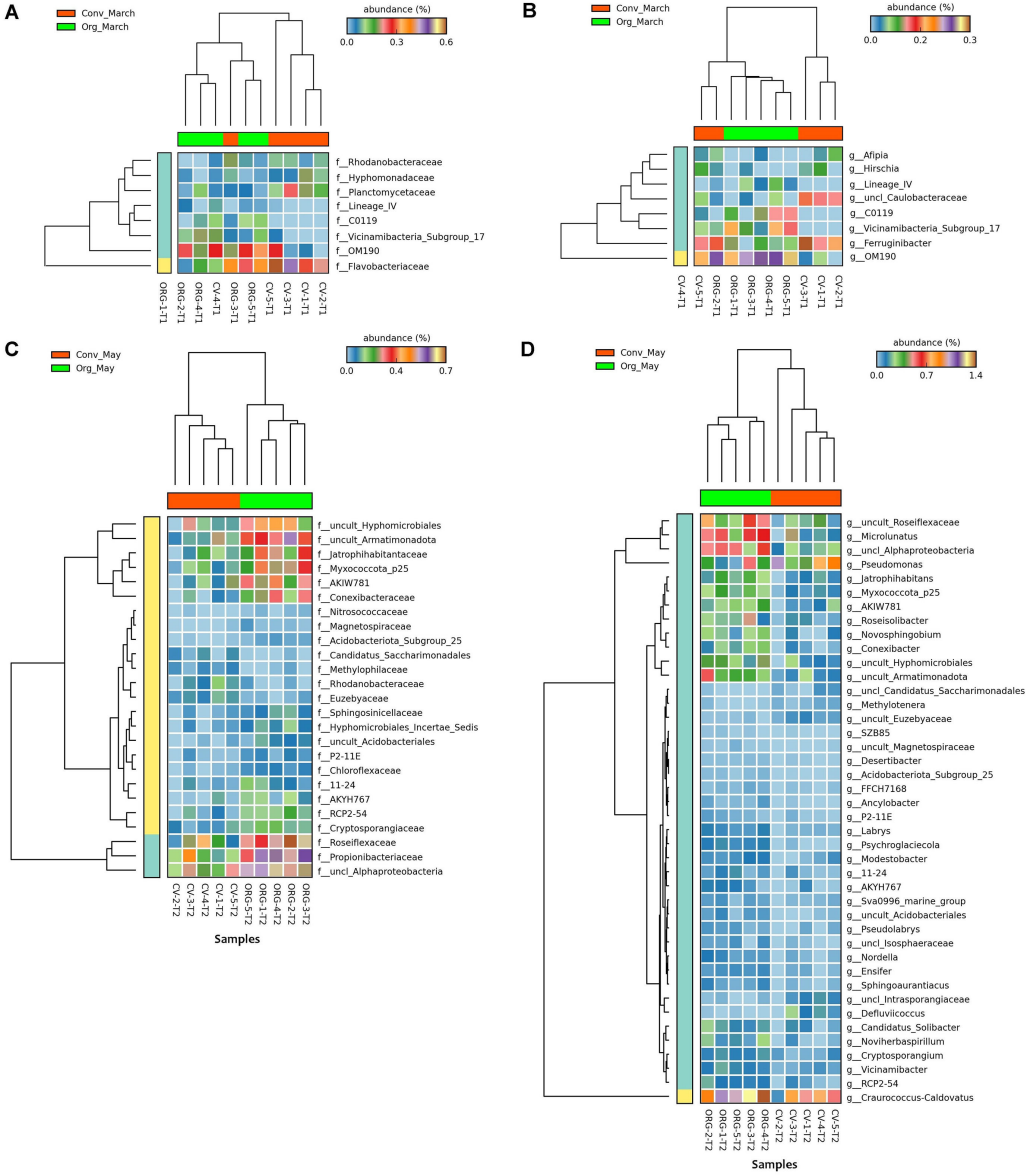
Differential ASV profiles between the conventional and organic wheat, in the March and May samplings, at the family **(A, C)** and genus **(B, D)** taxonomic levels.

**Figure 6:**
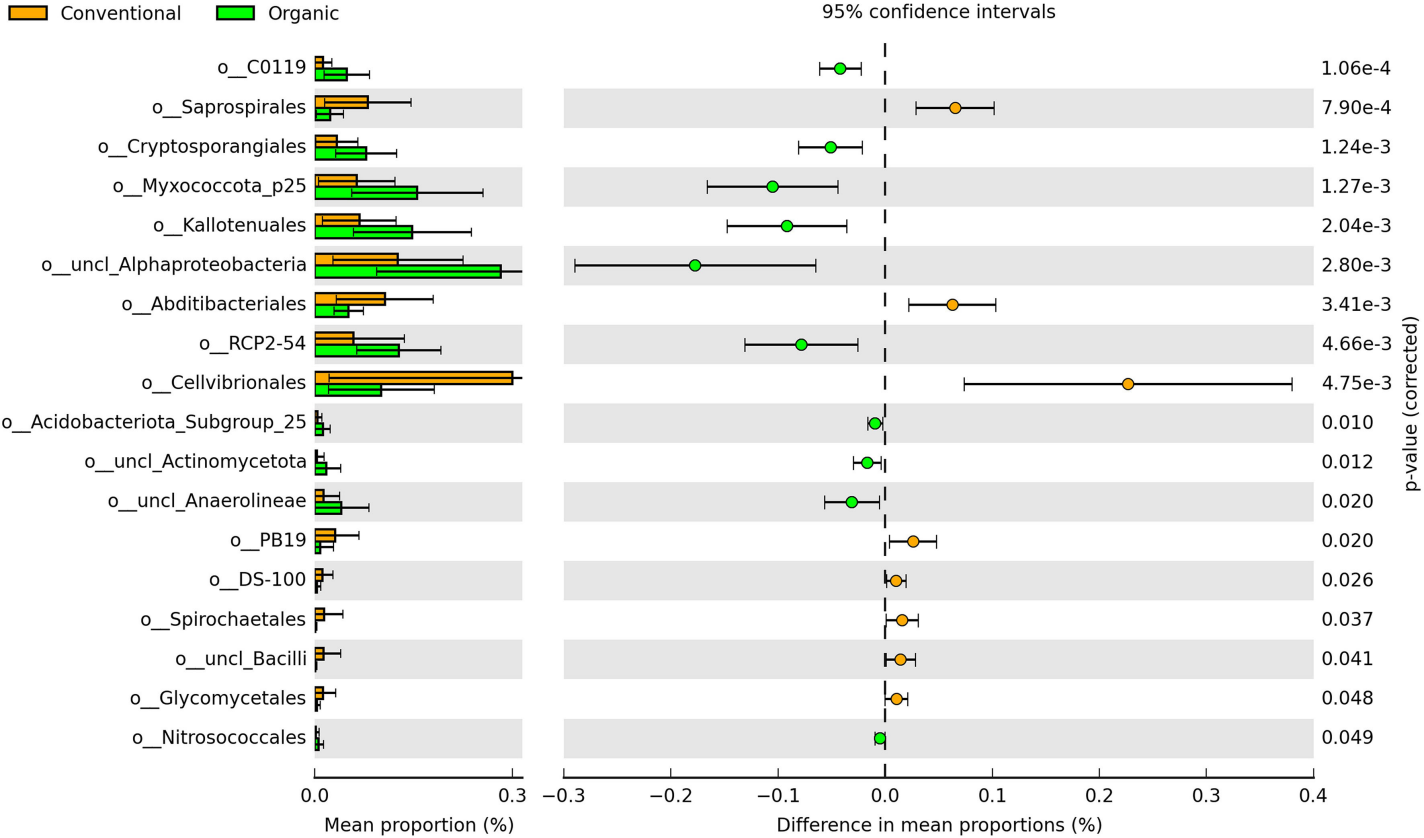
Differential representation of most abundant ASV profiles comparing the conventional with the organic wheat fields (all samples).

In general, a shift towards a richer metabarcoding profile was observable in May for both fields, with a higher number of ASVs that were more abundant in organic samples, both at the genus and family levels (**Figures 5C, D**), showing distinct clusters separated by sampling time in the conventional field (**Figures 5A–D**). When the sampling time was not considered in the comparisons, Cellvibrionales, Saprospirales, Abditibacteriales, and PB019 were more represented in the conventional crop, with remaining orders, including uncl. Alphaproteobacteria, again more abundant in the organic field samples (**Figure 6**).

**Figure 7:**
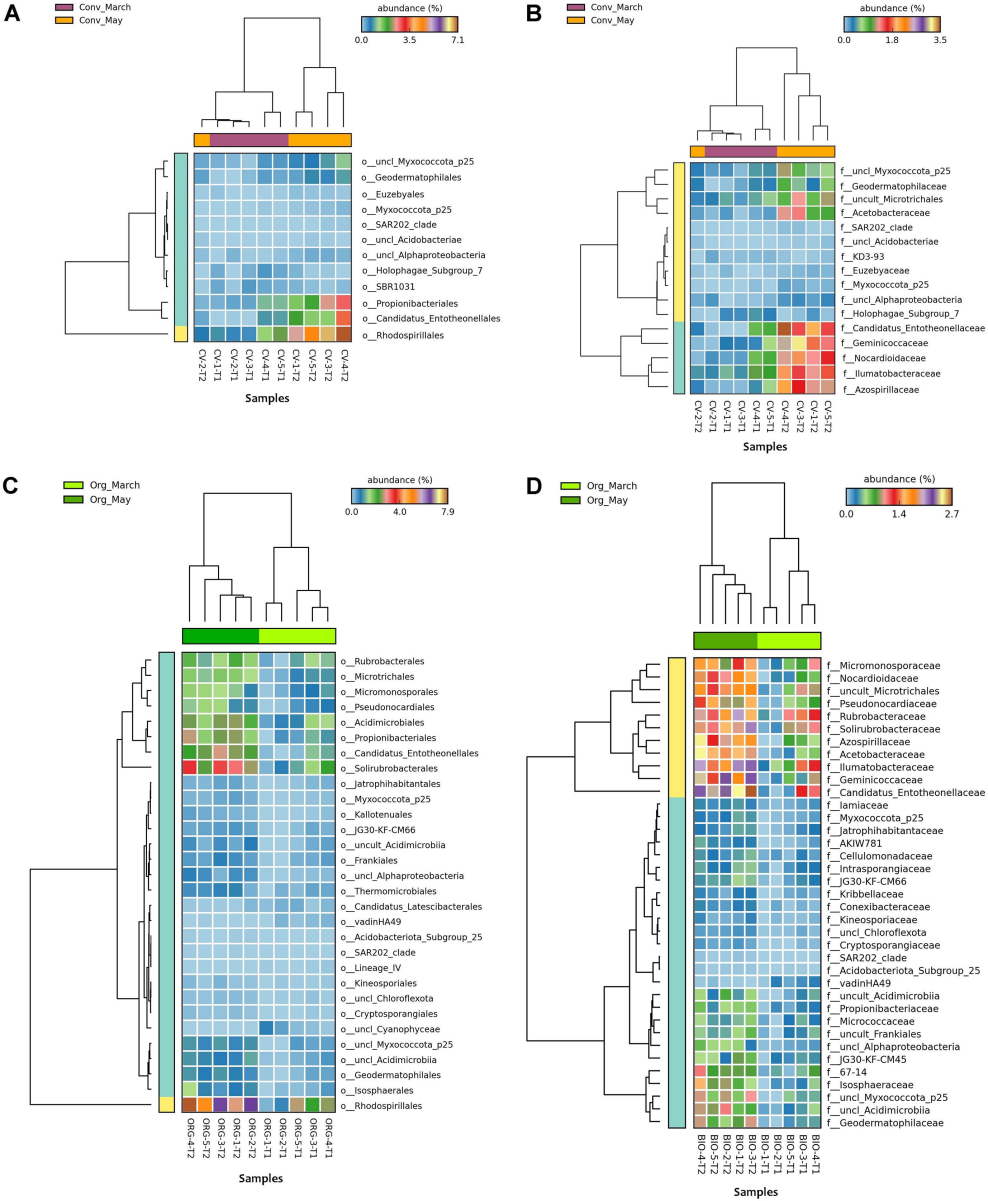
Differential representation of most abundant ASV metabarcoding profiles observed, shown at the order and family levels and by sampling time, in the conventional **(A, B)** and organic **(C, D)** wheat fields.

As shown by PERMANOVA, a significant effect was also observed when comparing ASV metabarcoding profiles by season, in both the control and cultivated sample sets. The taxa clustered, at the order or family levels, in distinct groups, congruent with the two sampling times, with the organic wheat showing again a higher number of taxa (**Figures 7A–D**). This richness was also reflected at the genus level, with the organic crop showing more than twice the differentially represented ASVs found in the conventional field (**Supplementary Figure 5**).

PERMANOVA showed few significant effects for the nematode abundance when classifying the samples by feeding groups, except for the population prevalence of fungal/moss feeders (*p* = 0.042, **Supplementary Table 4**). The bacterial taxa most represented in samples with low prevalence of these nematodes were *Pirellula* sp., with other unclassified members of Pedosphaeraceae, Acidimicrobiia, or TRA3-20 (**Supplementary Figure 6**).

Significant differences in ASV abundance and profiles were also found when comparing samples grouped by each cultivation type with their corresponding controls. The two management types showed distinct groups of differentially represented ASV, together with a seasonal effect, as shown by the distinct profiles observed between the two sampling times. The differentially represented ASVs were, at both sampling times, mostly more abundant in the controls. Moreover, the profiles differed between the two cultivation types, with the only exception of uncl. Pedospherae (p_Verrucomicrobia) in common between the two systems (**Supplementary Figures 7A, B**).

### ASV and nematodes

**Figure 8:**
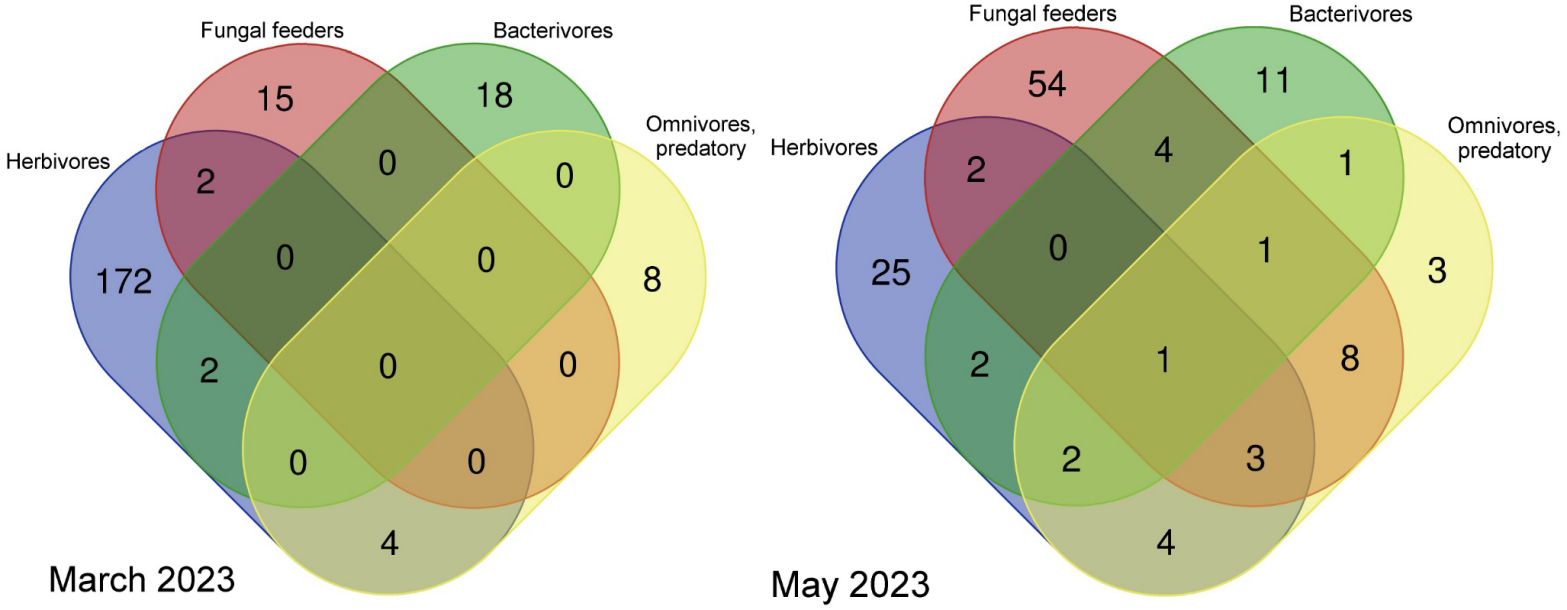
Venn diagrams indicating the number of ASV with a significant Spearman’s correlation (*p* < 0.05) of sequence abundance with the densities of nematodes classified in four different guilds, at both sampling times (pooled samples).

We also investigated the relationships of bacteria with nematodes, in order to highlight any potential for nematode regulation or other possible services carried out in the rhizosphere. Pooled sample data showed that the ASV responses to nematodes were specific for each guild, as shown by significant Spearman’s correlations of bacteria originating groups, characterized by variable compositions. Venn diagrams showed that these associations, when ordered for each variable by decreasing *p*-values, originated groups of correlated ASV specific for each guild (**Figure 8**). If in common between two guilds, the ASV often showed opposite correlations. The most significant (top 10) correlations with herbivores were shown in March by ASVs that were mostly unclassified, including a member of Pedosphaeraceae. The most significant negative correlations were shown in March by members of Cyanophyceae, *Cylindrospermum* sp., *Methylobacterium/Methylorubrum*, *Sphingomonas paucimobilis*, and a *Candidatus* member of Saccharimonadales. The top 10 correlations observed in May were mostly negatives, involving, i.e., unclassified members of Pirellulaceae; *Chryseolinea* sp.; members of Sphingobacteriaceae, Microscillaceae, and Geminicoccaceae; and *Nitrospira japonica* (**Supplementary Table 6**).

### ASV and soil profiles

After filtering the ASV with >30 null values, main linear correlations showed a community subset of bacteria with sensitivity to the number of nematode taxa (124 ASV with negative correlations vs. 6 positive) and the soil electric conductivity (124 negative correlations vs. 19 positive). Most significant links of ASV occurred with the total inorganic C (146 positive correlations vs. 7 negative) and the AP content of soil (237 positive correlations vs. 7 negative). The remaining variables (pH, organic C, C/N, and N content) showed positive and negative correlations in the same order of magnitude (range = 7–80) (**Supplementary Table 7**).

The top 10 most abundant ASV showed lineages with multiple or specific metabolic properties, including an uncl. *Methylobacterium/Methylorubrum* sp., *Sphingomonas paucimobilis*, with unclassified or uncultured members of Vicinamibacterales, Gemmatimonadaceae, Entotheonellaceae, Comamonadaceae, and BIrii_41 (**Table 3**).

**Table 3:** Pearson’s correlations of the top 10 most abundant ASV with soil variables and main properties ^1^.

Taxa	Variable^2^	*r*	*P-*value	Main traits
*Methylobacterium*/*Methylorubrum* sp.	TIC	−0.3921	0.01	Phyllosphere-associated, soil and water inhabitant, lignin and aromatic compound degradation, nematode antagonism (Grossi et al., 2025)
*Sphingomonas paucimobilis*	TIC	−0.4135	0.01	In low-nutrient environments, soil and water, opportunistic clinical pathogen, hetero- and oligo-trophic, biofilms (Steinberg and Burd, 2015)
uncult. Vicinamibacterales_4	P	0.4180	0.001	Organic matter decomposers (Huber and Overmann, 2019)
uncl. Vicinamibacteraceae_1	P	0.4827	0.001	Neutrophilic, psychrotolerant to mesophilic chemoheterotrophs (Huber and Overmann, 2019)
uncl. Vicinamibacteraceae_2	P	0.3774	0.05	Neutrophilic, psychrotolerant to mesophilic chemoheterotrophs (Huber and Overmann, 2019)
uncl_Gemmatimonadaceae	P	0.3308	0.05	Polyphosphate-accumulating (Zhang et al., 2003)
uncl. Entotheonellaceae_1	N. taxa	−0.3456	0.05	Marine symbionts, producers of anticancer metabolites (Brück et al., 2008)
uncl. Entotheonellaceae_1	OC	−0.3744	0.05	
uncl. Entotheonellaceae_1	N	−0.3560	0.05	
uncl. Entotheonellaceae_1	CE	−0.4723	0.001	
uncl. Entotheonellaceae_1	pH	−0.3614	0.05	
uncl. Comamonadaceae	TIC	0.5289	0.001	Organotrophs, anaerobic denitrifiers, iron-reducing, hydrogen oxidizers, photoautotrophic and photo-heterotrophic, fermentative bacteria (Willems, 2014)
uncl. Comamonadaceae	P	0.4210	0.001	
BIrii 41_3 (Polyangiales, Myxococcota, Deltaproteobacteria)	CE	−0.3693	0.05	A lineage of predatory bacteria (Dai et al., 2023)

^1^ March, all samples. Calculated with PAST, filtered ASV dataset (619 ASV).

^2^ TIC, total inorganic C; N. taxa, nematode taxa per sample; CE, soil electric conductivity.

## Discussion

### Soil profiles and nematodes

Our study aimed to evaluate, at two sampling times, the effects of organic and conventional wheat crop types on the diversity of nematodes and rhizosphere bacteria, considering non-cultivated background controls as reference, and soil physicochemical properties. The soil and climate similarities and the plant genotype identity reduced the source of variability among samples and allowed focusing on wheat cultivation and cropping systems. Fertilization and sampling time appeared as the most effective drivers, affecting some soil physicochemical traits, with changes in nematode populations. This effect was also shown by PCA, which separated samples according to cultivation and soil management type. Fertilization appeared as the main factor accounting for the differences among treatments. Different studies reported the effect of NH_4_NO_3_ fertilization on soil through nutrient and element enrichment, also affecting soil physicochemical properties (Bouman et al., 1995**;** Wei et al., 2012**;** Cui et al., 2024). However, the organic wheat soil, relying only on foliar fertilization, showed a pH that is slightly but significantly lower than the conventional field (6.97 vs. 7.18, respectively; *p* = 0.013, **Supplementary Table 1b**), an effect likely due to the application method of fertilizers since organically cultivated wheat plants could not rely on a basal application of fertilizer as in conventional farming; they have had to increase the acidification of the rhizosphere to recover nutrients.

Consistent with our observations, nitrogen fertilization also negatively affected the number of plant parasitic nematodes, an effect observed for NH_4_NO_3_ and grassland nematode communities, and ascribed to ammonia toxicity (Zhou et al., 2021**;** Wei et al., 2012). The lower herbivore densities found in both the conventional and organic wheat fields, compared with the uncultivated controls (**Figure 1**), also reflected the monoculture cropping intensification. In general, the nematode guilds showed specific and consistent trends in time, with the herbivores most represented in both controls. Plant richness and diversity, as those found in the control samples, are known to increase the densities and diversity of nematodes, owing to a higher diversity of food sources. A higher plant biomass, as found in the wheat monocultures, may have instead reduced the numbers of herbivores, because of regulation effects (Dietrich et al., 2021).

The nematode population compositions also reflected the differences in the cropping technologies applied. In the organic wheat field, and at both sampling times, the numbers of fungal/moss feeders were higher than the herbivores, with an opposite situation found in the conventional crop, likely indicating an effect of the fungicides applied in the conventional system. Seasonal changes were also detected among nematode feeding groups, mostly characterized by the inverse relationship of herbivores with the other groups in March, with a shift between sampling times. In May, herbivores switched toward a positive relationship with bacterivores, also showing a stronger correlation with the omnivores/predators, reflecting factors such as regulation and/or links to higher multiplication rates (i.e., due to higher root biomass availability and milder temperatures), including the appearance of less represented nematode taxa.

### ASV metabarcoding profiles

Sampling on the same wheat variety allowed us to identify crop management type, sampling time, and soil physicochemical traits as significant factors affecting the rhizosphere microbiota. Plant genotype, together with field spatial variability, was reported to affect the rhizosphere microbiota. In particular, when comparing modern with ancient wheat varieties, a higher bacterial diversity was reported in modern genotypes, with qualitative changes in microbiota and PGPR profiles (Reid et al., 2024). An effect of crop inputs on microbial diversity was also reported for, i.e., the application of fertilizers and herbicides (Jacquiod et al., 2022). Consistent with these reports, the conventional wheat in our study showed a low diversity in March (as shown by Berger–Parker and evenness indexes), compared with both uncultivated controls, and associated with a higher dominance of a few taxa. However, this effect was not evident in May, when only the organic crop showed a higher richness of species but only when compared with the organic control, likely due to the wheat biomass (**Supplementary Table 2**). The four treatments also showed similar β-diversity levels, as indicated by the large fraction of ASV in common between the two samplings, that decreased between the two sampling times, with the only exception of the conventional control, which in May was enriched in unique taxa (**Table 2**, **Figure 2**).

Alphaproteobacteria, including members of Methylobacteriaceae and Sphingomonadaceae, were the most represented lineages in the wheat crops (**Figure 3B**). Methylotrophic bacteria include diverse and partially described lineages of plant-associated and phyllosphere taxa, common in soil and fresh waters, that metabolize single, C_1_ sources (Leducq et al., 2022). They are known for several beneficial services deployed in their microhabitats, with three distinct phyllosphere groups characterized by plant growth stimulation and protection, sequestration of contaminants, and N_2_ fixation capabilities (Madhaiyan et al., 2007**;** Leducq et al., 2022). *Methylobacterium* spp. play effective roles in the phyllosphere, including the degradation and demethoxylation of lignin and related aromatic compounds, such as vanillate, present in the root exudates. These bacteria are also involved in root signaling and nodulation processes (Lee et al., 2022**;** Ehinmitan et al., 2025). Taxa reported as phyllosphere-associated include *Methylobacterium adhaesivum*, known for adhesive and biofilm-production properties, enriched in unfertilized wheat when compared with fertilized plants (Cui et al., 2024).

Members of Sphingomonadaceae include various PGPR or endophytic species characterized by key metabolic capabilities such as the degradation of phenolic compounds and P metabolism. In our study, *Sphingobium* sp. and further unclassified taxa were correlated with the P content of soil. *Novosphingobium* sp. inverse correlations with the soil N content and EC likely reflected the growth capability of these bacteria in low N and nutrient conditions (**Supplementary Table 7**).

The ASV profiles also differed by management type and sampling times. When comparing samples by system management, the differences indicated the occurrence of a higher level of redundancy in the organic field. Different ASVs were involved, in the two systems, in services such as organic matter decomposition and nutrient recycling. However, the organic field showed in March a higher number of taxa, including the more represented members of OM190, C0119, and Vicinamibacteria, whereas the conventional field showed only a higher representation of Saprospirales in March (**Figure 4A**), and Euzebyales in May (**Figure 4B**). Members of OM190 are involved in polysaccharides and organic matter degradation. They were reported as dominant in straw-enriched, unfertilized cereal systems, as well as in association with microalgae (Behnke et al., 2021**;** Lage and Bondoso, 2014). Similarly, C0119 taxa, also active in organic matter degradation, became dominant in paddy rice soil emended with biochar (Tang et al., 2021). In addition, some Vicinamibacteria, considered as beneficial soil species utilizing complex C sources and involved in soil health and balance, contribute to the organic matter decomposition (Huber and Overmann, 2019). Finally, a number of Saprospirales are involved in the hydrolysis and degradation of organic matter and complex organic compounds (McIlroy and Nielsen, 2014). The higher diversity found in the organic field was hence reflected in a higher redundance of species available for the same (or similar) services provided, with a benefit derivable for the organic crop in terms of soil stability and system resilience.

Sampling time also affected the ASV profiles. The metabarcoding composition of both fields changed in May, showing a higher functional diversity of species, including predatory bacteria (Myxococcota), PGPR (Armatimonadota, Alphaproteobacteria, and Cryptosporangiales), and taxa involved in nutrient cycling (RCP2-54), cellulose, carbohydrate, and amino acid metabolism (Kallotenuales and Jatrophihabitantales). Together with photoautotrophs (Chloroflexales), these taxa were more represented in the organic system (**Figures 5C, D**). The shift toward a higher number of ASVs in May originated distinct clusters separating the two systems also at lower taxonomic levels, with a consistent, higher abundance of Alphaproteobacteria in the organic field (**Figures 5**, **6**).

In conclusion, the sampling season induced significant changes in the bacterial community, with the organic wheat always characterized by a higher richness and redundancy. This is consistent with other studies reporting seasonal variations of environmental variables, such as temperature, moisture, and nutrient availability, as the main drivers of microbial profiles (Robinson et al., 2016**;** Kim et al., 2022**;** Yin et al., 2024).

### Crops vs. controls

Differences between the two management types were reflected in the differentially represented ASVs found when comparing the wheat crops with their respective controls. This comparison also confirmed the occurrence of a seasonal effect, as shown by the changes observed between the two sampling times. Controls showed a higher ASV abundance at both sampling times, with metabolic traits of the bacteria involved that differed between the two cultivation types (**Supplementary Figures 7A, B**). In March, the ASV more represented in the organic control differed from those of the conventional control, being characterized by taxa with a metabolic versatility including oligotrophic metabolism, production of bioactive secondary metabolites, or degradation of aromatic compounds. The ASV more represented in the conventional field control showed instead specialized taxa involved in processes such as polysaccharide degradation and glycolysis, together with predation (i.e., o_Nannocystales). The differentially represented ASV found in May in the organic field, differing from the March samplings, showed taxa involved in C_1_ methylotrophic metabolism, N_2_ fixation (Hyphomicrobiales), and lignin and carbohydrate decomposition (Sphingobacteriales). Chitin-degrading bacteria (*Ca*. “Sumerlaeales”), sugar/carbohydrate polymer users (Trueperales and Acidobacteriae), and rare taxa (Euzebiales) were the more represented taxa in the conventional field (**Supplementary Figures 7A, B**).

### ASV and nematodes

The most significant interaction of ASV with nematode feeding groups involved the fungal/moss feeders, with *Pirellula* sp., uncl. Pedosphaeraceae, Acidimicrobiia, or TRA3–20 as enriched in samples with a low prevalence of these nematodes. Although these taxa are not previously known to be directly involved in nematode regulation, their higher ASV representation may reflect direct or indirect interactions with the nematode’s rhizosphere activity and/or with other bacteria or microfaunal taxa. However, more information on the links between bacteria and nematodes could be retrieved by analyzing Spearman’s correlations between ASV sequence abundance and nematode densities, classified in the four guilds. The ASV–guild associations appeared specific, with groups of bacteria showing significant correlations that were unique for a specific guild. The herbivores and the fungal feeders were the groups showing the highest numbers of significant correlations, in March and May, respectively. In May, most ASV correlations with herbivores or bacterivores were negative (68.9% and 95.4%, respectively). Bacteria negatively correlated with herbivores included taxa known for nematode antagonism such as *Bacillus* sp. or *Methylorubrum* sp., a PGPR also reported as antagonistic vs. root-knot nematodes (Prhabu et al., 2009**;** Zhao et al., 2023). Further taxa included unclassified Lactobacillales, Cyanobacteria, *Mycoplasma* spp., and other pathogenic bacteria. Positive correlations included PGPR, and taxa involved in N_2_ and nutrient cycling, denitrification, and ammonia oxidation, such as *Rhizobium*, *Nitrospira*, and *Nitrosomonas*. The occurrence of bacteria selectively associated with each feeding guild is consistent with studies on nematodes–bacteria associations in tropical and subtropical crops (Colagiero et al., 2025) and with the occurrence of a complex web of functional services acting at the nematode–bacteria–rhizosphere interface (Trejo-Meléndez and Contreras-Garduño, 2025).

## Data Availability

The datasets presented in this study can be found in online repositories. The names of the repository/repositories and accession number(s) can be found in the article/**Supplementary Material**.

## Footnotes

1 ISO 17892-4:2016. Geotechnical investigation and testing — Laboratory testing of soil — Part 4: Determination of particle size distribution.

2 ISO 10694:1995. Soil quality — Determination of organic and total carbon after dry combustion (elementary analysis)

## References

[B1] Acuña J. J. Rilling J. I. Inostroza N. G. Manquian J. Zhang Q. Gupta V. V. S. R. . (2023). Diversity, community structure, and potential functions of root-associated bacterial communities of different wheat (Triticum aestivum) cultivars under field conditions. Agronomy 13, 1392. doi: 10.3390/agronomy13051392 30654563

[B2] Anderson M. Habiger J. (2012). Characterization and identification of productivity-associated rhizobacteria in wheat. Appl. Envir. Microbiol. 78, 4434–4446. doi: 10.1128/AEM.07466-11 22504815 PMC3370509

[B3] Anikwe M. A. N. Ife K. (2023). The role of soil ecosystem services in the circular bioeconomy. Front. Soil Sci. 3, 1209100. doi: 10.3389/fsoil.2023.1209100

[B4] Azarbad H. Bainard L. D. Agoussar A. Tremblay J. Yergeau E. (2022). The response of wheat and its microbiome to contemporary and historical water stress in a field experiment. ISME Commun. 2, 62. doi: 10.1038/s43705-022-00151-2 37938737 PMC9723694

[B5] Baer S. G. Birgé H. E. (2018). “ Soil ecosystem services: an overview,” in Managing Soil Health for Sustainable Agriculture. Vol. 1: Fundamentals. Ed. Reicosky D. ( Burleigh Dodds Science Publishing, Cambridge, UK). doi: 10.19103/AS.2017.0033.02

[B6] Behnke G. D. Kim N. Zabaloy M. C. Riggins C. W. Rodriguez-Zas S. Villamil M. B. (2021). Soil microbial indicators within rotations and tillage systems. Microorganisms 9, 1244. doi: 10.3390/microorganisms9061244 34201118 PMC8228827

[B7] Bolyen E. Rideout J. R. Dillon M. R. Bokulich N. A. Abnet C. C. Al-Ghalith G. A. . (2019). Reproducible, interactive, scalable and extensible microbiome data science using QIIME 2. Nat. Biotechnol. 37, 852–857. doi: 10.1038/s41587-019-0209-9 31341288 PMC7015180

[B8] Bouman O. T. Curtin D. Campbell C. A. Biederbeck V. O. Ukrainetz H. (1995). Soil acidification from long-term use of anhydrous ammonia and urea. Soil Sc. Soc Am. J. 59, 1488–1494. doi: 10.2136/sssaj1995.03615995005900050039x

[B9] Brück W. Sennett S. Pomponi S. Willenz P. McCarthy J. (2008). Identification of the bacterial symbiont Entotheonella sp. in the mesohyl of the marine sponge Discodermia sp. ISME J. 2, 335–339. doi: 10.1038/ismej.2007.91 18256706

[B10] Brussaard L. (2013). “ Ecosystem services provided by the soil biota,” in Soil Ecology and Ecosystem Services. Eds. Wall D. H. Bardgett R. D. Behan-Pelletier V. Herrick J. E. Jones H. Ritz K. ( Oxford University Press, UK), 45–58.

[B11] Callahan B. J. McMurdie P. J. Rosen M. J. Han A. W. Johnson A. J. A. Holmes S. P. (2016). DADA2: High-resolution sample inference from Illumina amplicon data. Nat. Meth. 13, 581–583. doi: 10.1038/nmeth.3869 27214047 PMC4927377

[B12] Caporaso J. G. Lauber C. L. Walters W. A. Berg-Lyons D. Lozupone C. A. Turnbaugh P. J. . (2011). Global patterns of 16S rRNA diversity at a depth of millions of sequences per sample. Proc Natl Acad Sci U S A. 108 Suppl 1, 4516–22. doi: 10.1073/pnas.1000080107 20534432 PMC3063599

[B13] Chen J. Sharifi R. Khan M. S. S. Islam F. Bhat J. A. Kui L. . (2022). Wheat microbiome: structure, dynamics, and role in improving performance under stress environments. Front. Microbiol. 12, 821546. doi: 10.3389/fmicb.2021.821546 35095825 PMC8793483

[B14] Clarridge J. E. (2004). Impact of 16S rRNA gene sequence analysis for identification of bacteria on clinical microbiology and infectious diseases. Clin. Microbiol. Rev. 17, 840–862. doi: 10.1128/CMR.17.4.840-862.2004 15489351 PMC523561

[B15] Colagiero M. Pocasangre L. Ciancio A. Pentimone I. Rosso L. C. (2025). Farming system and nematodes affect the rhizosphere microbiome of tropical banana plants. Environ. Microbiol. Rep. 17, e70155. doi: 10.1111/1758-2229.70155 40635331 PMC12241448

[B16] Cui C. Li F. Zeng Q. Li C. Shen W. Gao X. . (2024). Influence of fertilization methods and types on wheat rhizosphere microbiome community and functions. J. Agric. Food. Chem. 72, 7794–7806. doi: 10.1021/acs.jafc.3c09941 38561246

[B17] Curtis T. Halford N. G. (2014). Food security: the challenge of increasing wheat yield and the importance of not compromising food safety. Ann. Appl. Biol. 164, 354–372. doi: 10.1111/aab.12108 25540461 PMC4240735

[B18] Dai W. Liu Y. Yao D. Wang N. Ye X. Cui Z. . (2023). Phylogenetic diversity of stochasticity-dominated predatory myxobacterial community drives multi-nutrient cycling in typical farmland soils. Sc. Tot. Envir. 871, 161680. doi: 10.1016/j.scitotenv.2023.161680 36682558

[B19] Dietrich P. Cesarz S. Liu T. Roscher C. Eisenhauer N. (2021). Effects of plant species diversity on nematode community composition and diversity in a long-term biodiversity experiment. Oecologia 197, 297–311. doi: 10.1007/s00442-021-04956-1 34091787 PMC8505370

[B20] Djotan A. K. G. Matsushita N. Fukuda K. (2023). Paired root-soil samples and metabarcoding reveal taxon-based colonization strategies in arbuscular mycorrhizal fungi communities in Japanese cedar and cypress stands. Microb. Ecol. 86, 2133–2146. doi: 10.1007/s00248-023-02223-9 37115261 PMC10497666

[B21] Drake L. E. Cuff J. P. Young R. E. Marchbank A. Chadwick E. A. Symondson W. O. C. (2022). An assessment of minimum sequence copy thresholds for identifying and reducing the prevalence of artefacts in dietary metabarcoding data. Meth. Ecol. Evol. 13, 694–710. doi: 10.1111/2041-210X.13780 40046247

[B22] Dueholm M. K. D. Andersen K. S. Korntved A. K. C. Rudkjøbing V. Alves M. Bajón-Fernández Y. . (2024). MiDAS 5: Global diversity of bacteria and archaea in anaerobic digesters. Nat. Commun. 15, 5361. doi: 10.1038/s41467-024-49641-y 38918384 PMC11199495

[B23] Ehinmitan E. Siamalube B. Losenge T. Mamati E. Juma P. Ngumic V. (2025). Methylobacterium spp. in sustainable agriculture: strategies for plant stress management and growth promotion. Microbe 8, 100476. doi: 10.1016/j.microb.2025.100476

[B24] Enghiad A. Ufer D. Countryman A. M. Thilmany D. D. (2017). An overview of global wheat market fundamentals in an era of climate concerns. Int. J. Agron. 2017, 3931897. doi: 10.1155/2017/3931897

[B25] Erenstein O. Jaleta M. Mottaleb K. A. Sonder K. Donovan J. Braun H. J. (2022). “ Global trends in wheat production, consumption and trade,” in Wheat Improvement. Eds. Reynolds M. P. Braun H. J. ( Springer, Cham). doi: 10.1007/978-3-030-90673-3_4

[B26] Fouad N. El-Zayat E. M. Amr D. El-Khishin D. A. Abd-Elhalim H. M. Hafez A. . (2025). Characterizing wheat rhizosphere bacterial microbiome dynamics under salinity stress: insights from 16s rRNA metagenomics for enhancing stress tolerance. Plants 14, 1033. doi: 10.3390/plants14071033 40219101 PMC11990312

[B27] Garrido-Sanz D. Keel C. (2025). Seed-borne bacteria drive wheat rhizosphere microbiome assembly via niche partitioning and facilitation. Nat. Microbiol. 10, 1130–1144. doi: 10.1038/s41564-025-01973-1 40140705 PMC12055584

[B28] Giannelli G. Del Vecchio L. Cirlini M. Gozzi M. Gazza L. Galaverna G. . (2024). Exploring the rhizosphere of perennial wheat: potential for plant growth promotion and biocontrol applications. Sci. Rep. 14, 22792. doi: 10.1038/s41598-024-73818-6 39354104 PMC11445523

[B29] Göker M. Christensen H. Fingerle V. Kostovski M. Margos G. Moore E. R. B. . (2025). List of recommended names for bacteria of medical importance: report of the ad hoc committee on mitigating changes in Prokaryotic Nomenclature. Int. J. Syst. Evol. Microbiol. 75, 6943. doi: 10.1099/ijsem.0.006943 41129200 PMC12548757

[B30] Grossi C. E. M. Ulloa R. M. Sahin N. Tani A. (2025). Methylobacterium as a key symbiont in plant-microbe interactions: its ecological and agricultural significance. Pl. Bio/Technol. 42, 229–241. doi: 10.5511/plantbiotechnology.25.0309a 41181079 PMC12573535

[B31] Grote U. Fasse A. Nguyen T. T. Erenstein O. (2014). Food security and the dynamics of wheat and maize value chains in Africa and Asia. Front. Sustai. Food. Sys. 4, 617009. doi: 10.3389/fsufs.2020.617009

[B32] Hammer Y. Harper D. A. T. Ryan P. D. (2001). PAST: Paleontological statistics software package for education and data analysis. Paleont. El. 4, 4. doi: 10.1002/9781119933960 41531421

[B33] Heberle H. Meirelles G. V. da Silva F. R. Telles G. P. Minghim R. (2015). InteractiVenn: a web-based tool for the analysis of sets through Venn diagrams. BMC Bioinf. 16, 169. doi: 10.1186/s12859-015-0611-3 25994840 PMC4455604

[B34] Huber K. J. Overmann J. (2019). “ Vicinamibacter,” in Bergey’s Manual of Systematics of Archaea and Bacteria ( John Wiley & Sons, in association with Bergey’s Manual Trust). doi: 10.1002/9781118960608.gbm01685

[B35] Jacquiod S. Raynaud T. Pimet E. Ducourtieux C. Casieri L. Wipf D. . (2022). Wheat rhizosphere microbiota respond to changes in plant genotype, chemical inputs, and plant phenotypic plasticity. Front. Ecol. Evol. 10, 903008. doi: 10.3389/fevo.2022.903008

[B36] Jalili V. Afgan E. Gu Q. Clements D. Blankenberg D. Goecks J. . (2020). The Galaxy platform for accessible, reproducible and collaborative biomedical analyses: 2020 update. Nucleic Acids Res. 48, W395–W402. doi: 10.1093/nar/gkaa434 32479607 PMC7319590

[B37] Kavamura V. N. Mendes R. Bargaz A. Mauchline T. H. (2021). Defining the wheat microbiome: towards microbiome-facilitated crop production. Comp. Struct. Bio/Technol. J. 19, 1200–1213. doi: 10.1016/j.csbj.2021.01.045 33680361 PMC7902804

[B38] Kawasaki A. Dennis P. G. Forstner C. Raghavendra A. K. H. Richardson A. E. Watt M. . (2021). The microbiomes on the roots of wheat (Triticum aestivum L.) and rice (Oryza sativa L.) exhibit significant differences in structure between root types and along root axes. Funct. Plant Biol. 48, 871–888. doi: 10.1071/FP20351 33934748

[B39] Kim S. Y. Zhou X. Freeman C. Kang H. (2022). Changing thermal sensitivity of bacterial communities and soil enzymes in a bog peat in spring, summer and autumn. Appl. Soil Ecol. 173, 104382. doi: 10.1016/j.apsoil.2021.104382 38826717

[B40] Kinnunen-Grubb M. Sapkota R. Vignola M. Marques Nunes I. Nicolaisen M. (2020). Breeding selection imposed a differential selective pressure on the wheat root-associated microbiome. FEMS Microbiol. Ecol. 96, fiaa196. doi: 10.1093/femsec/fiaa196 32970821

[B41] Kokalis-Burelle N. Kloepper J. W. Reddy M. S. (2006). Plant growth-promoting rhizobacteria as transplant amendments and their effects on indigenous rhizosphere microorganisms. Appl. Soil Ecol. 31, 91–100. doi: 10.1016/j.apsoil.2005.03.007 38826717

[B42] Lage O. M. Bondoso J. (2014). Planctomycetes and macroalgae, a striking association. Front. Microbiol. 5, 267. doi: 10.3389/fmicb.2014.00267 24917860 PMC4042473

[B43] Leducq J. B. Sneddon D. Santos M. Condrain-Morel D. Bourret G. Martinez-Gomez N. C. . (2022). Comprehensive phylogenomics of Methylobacterium reveals four evolutionary distinct groups and underappreciated phyllosphere diversity. Genome Biol. Evol. 14, evac123. doi: 10.1093/gbe/evac123 35906926 PMC9364378

[B44] Lee J. A. Stolyar S. Marx C. J. (2022). Aerobic methoxydotrophy: growth on methoxylated aromatic compounds by Methylobacteriaceae. Front. Microbiol. 13, 849573. doi: 10.3389/fmicb.2022.849573 35359736 PMC8963497

[B45] Leff J. W. Fierer N. (2013). Bacterial communities associated with the surfaces of fresh fruits and vegetables. PloS One 8, e59310. doi: 10.1371/journal.pone.0059310 23544058 PMC3609859

[B46] Liu Z. DeSantis T. Z. Andersen G. L. Knight R. (2008). Accurate taxonomy assignments from 16S rRNA sequences produced by highly parallel pyrosequencers. NAR 36, e120. doi: 10.1093/nar/gkn491 18723574 PMC2566877

[B47] Madhaiyan M. Poonguzhali S. Sa T. (2007). Metal tolerating methylotrophic bacteria reduces nickel and cadmium toxicity and promotes plant growth of tomato (Lycopersicon esculentum L.). Chemosphere 69, 220–228. doi: 10.1016/j.chemosphere.2007.04.017 17512031

[B48] McIlroy S. J. Nielsen P. H. (2014). “ The family saprospiraceae,” in The Prokaryotes: Other Major Lineages of Bacteria and the Archaea. Eds. Rosenberg E. Delong E. F. Lory S. Stackebrandt E. Thompson F. ( Springer, Heidelberg), 863–889.

[B49] Parks D. H. Tyson G. W. Hugenholtz P. Beiko R. G. (2014). STAMP: statistical analysis of taxonomic and functional profiles. Bioinformatics 30, 3123–3124. doi: 10.1093/bioinformatics/btu494 25061070 PMC4609014

[B50] Piombo E. Abdelfattah A. Droby S. Wisniewski M. Spadaro D. Schena L. (2021). Metagenomics approaches for the detection and surveillance of emerging and recurrent plant pathogens. Microorganisms 9, 188. doi: 10.3390/microorganisms9010188 33467169 PMC7830299

[B51] Prhabu S. Kumar S. Subramanian S. Sundaram S. P. (2009). Suppressive effect of Methylobacterium fujisawaense against root-knot nematode, Meloidogyne incognita. Ind. J. Nematol. 39, 165–169.

[B52] Quast C. Pruesse E. Yilmaz P. Gerken J. Schweer T. Yarza P. . (2013). The SILVA ribosomal RNA gene database project: improved data processing and web-based tools. Opens external link in new window. Nucl. Acids Res. 41, D590–D596. doi: 10.1093/nar/gks1219 23193283 PMC3531112

[B53] Quiza L. Tremblay J. Pagé A. P. Greer C. W. Pozniak C. J. Li R. . (2023). The effect of wheat genotype on the microbiome is more evident in roots and varies through time. ISME Commun. 3, 32. doi: 10.1038/s43705-023-00238-4 37076737 PMC10115884

[B54] R Core Team (2013). R: A Language and Environment for Statistical Computing (Vienna, Austria: R Foundation for Statistical Computing). Available online at: https://www.R-project.org/ (Accessed October 21, 2025).

[B55] Reid T. E. Kavamura V. N. Torres-Ballesteros A. Smith M. E. Abadie M. Pawlett M. . (2024). Agricultural intensification reduces selection of putative plant growth-promoting rhizobacteria in wheat. ISME J. 18, wrae131. doi: 10.1093/ismejo/wrae131 38990206 PMC11292143

[B56] Reinhold-Hurek B. Bünger W. Burbano C. S. Sabale M. Hurek T. (2015). Roots shaping their microbiome: global hotspots for microbial activity. Annu. Rev. Phytopathol. 53, 403–424. doi: 10.1146/annurev-phyto-082712-102342 26243728

[B57] Revelle W. (2017). Psych: Procedures for Personality and Psychological Research (Evanston, Illinois, USA: Northwestern University). Available online at: https://CRAN.R-project.org/package=psych (Accessed January 12, 2026).

[B58] Robinson R. J. Fraaije B. A. Clark I. M. Jackson R. Hirsch P. R. Mauchline T. H. (2016). Endophytic bacterial community composition in wheat (Triticum aestivum) is determined by plant tissue type, developmental stage and soil nutrient availability. Plant Soil 405, 381–396. doi: 10.1007/s11104-015-2495-4 30311153

[B59] Schippers B. Bakker A. W. Bakker P. A. H. M. (1987). Interactions of deleterious and beneficial rhizosphere microorganisms and the effect of cropping practices. Ann. Rev. Phytopathol. 25, 339–358. doi: 10.1146/annurev.phyto.25.1.339 41139587

[B60] Schiro G. Colangeli P. Müller M. E. H. (2019). Metabarcoding analysis of the mycobiome of wheat ears across a topographically heterogeneous field. Front. Microbiol. 10, 3389. doi: 10.3389/fmicb.2019.02095 31552005 PMC6746991

[B61] Schmidt R. L. Azarbad H. Bainard L. Tremblay J. Yergeau E. (2024). Intermittent water stress favors microbial traits that better help wheat under drought. ISME Commun. 4, ycae074. doi: 10.1093/ismeco/ycae074 38863723 PMC11165427

[B62] Sparks D. L. Page A. L. Helmke P. A. Loeppert R. H. Soltanpour P. N. Tabatabai M. A. . (1996). Methods of Soil Analysis. Part 3. Chemical Methods Vol. Vol. 5 (Madison: SSSA).

[B63] Steinberg J. P. Burd E. M. (2015). “ 238 - other gram-negative and gram-variable bacilli,” in Mandell, Douglas, and Bennett's Principles and Practice of Infectious Diseases (Eighth Edition). Eds. Bennett J. E. Dolin R. Blaser M. J. (Philadelphia: W.B. Saunders), 2667–2683.e4. doi: 10.1016/B978-1-4557-4801-3.00238-1

[B64] Sudermann M. A. Foster Z. S. L. Chang J. H. Grünwald N. J. (2024). Metabarcoding for plant pathologists. Can. J. Plant Pathol. 46, 142–160. doi: 10.1080/07060661.2023.2290041 37339054

[B65] Takahashi S. Tomita J. Nishioka K. Hisada T. Nishijima M. (2014). Development of a prokaryotic universal primer for simultaneous analysis of Bacteria and Archaea using Next-Generation Sequencing. PloS One 9, e 105592. doi: 10.1371/journal.pone.0105592 25144201 PMC4140814

[B66] Tang Z. Zhang L. He N. Gong D. Gao H. Ma Z. . (2021). Soil bacterial community as impacted by addition of rice straw and biochar. Sci. Rep. 11, 22185. doi: 10.1038/s41598-021-99001-9 34773058 PMC8589988

[B67] Timmusk S. Abd El-Daim I. A. Copolovici L. Tanilas T. Kännaste A. Behers L. . (2014). Drought-tolerance of wheat improved by rhizosphere bacteria from harsh environments: enhanced biomass production and reduced emissions of stress volatiles. PloS One 9, e96086. doi: 10.1371/journal.pone.0096086 24811199 PMC4014485

[B68] Trejo-Meléndez V. J. Contreras-Garduño J. (2025). Master of puppets: how microbiota drive the nematoda ecology and evolution? Ecol. Evol. 15, e71549. doi: 10.1002/ece3.71549 40837528 PMC12361815

[B69] Van de Peer Y. Chapelle S. De Wachter R. (1996). A quantitative map of nucleotide substitution rates in bacterial rRNA. NAR 24, 3381–3391. doi: 10.1093/nar/24.17.3381 8811093 PMC146102

[B70] Wei C. Zheng H. Li Q. Lü X. Yu Q. Zhang H. . (2012). Nitrogen addition regulates soil nematode community composition through ammonium suppression. PloS One 7, e43384. doi: 10.1371/journal.pone.0043384 22952671 PMC3432042

[B71] Wickham H. (2016). Ggplot2: Elegant Graphics for Data Analysis (New York: Springer-Verlag).

[B72] Willems A. (2014). “ The family comamonadaceae,” in The Prokaryotes. Eds. Rosenberg E. DeLong E. F. Lory S. Stackebrandt E. Thompson F. ( Springer, Berlin, Heidelberg). Available online at: doi: 10.1007/978-3-642-30197-1_238.

[B73] Xue C. Ryan Penton C. Shen Z. Zhang R. Huang Q. Li R. . (2015). Manipulating the banana rhizosphere microbiome for biological control of Panama disease. Sci. Rep. 5, 11124. doi: 10.1038/srep11124 26242751 PMC4525139

[B74] Xun W. Liu Y. Ma A. Yan H. Miao Y. Shao J. . (2024). Dissection of rhizosphere microbiome and exploiting strategies for sustainable agriculture. New Phytol. 242, 2401–2410. doi: 10.1111/nph.19697 38494698

[B75] Yang S. Lin S. Kelen G. D. Quinn T. C. Dick J. D. Gaydos C. A. . (2002). Quantitative multiprobe PCR assay for simultaneous detection and identification to species level of bacterial pathogens. J. Clin. Microbiol. 40, 3449–3454. doi: 10.1128/JCM.40.9.3449-3454.2002 12202592 PMC130696

[B76] Yin S. Wang C. Abalos D. Guo Y. Pang X. Tan C. . (2024). Seasonal response of soil microbial community structure and life history strategies to winter snow cover change in a temperate forest. Sc. Tot. Envir. 949, 175066. doi: 10.1016/j.scitotenv.2024.175066 39079633

[B77] Zhang H. Sekiguchi Y. Hanada S. Hugenholtz P. Kim H. Kamagata Y. . (2003). Gemmatimonas aurantiaca gen. nov. sp. nov. a gram-negative, aerobic, polyphosphate-accumulating micro-organism, the first cultured representative of the new bacterial phylum Gemmatimonadetes phyl. nov. Int. J. Syst. Evol. Microbiol. 53, 1155–1163. doi: 10.1099/ijs.0.02520-0 12892144

[B78] Zhao Z. Wang L. Khan R. A. A. Song X. Najeeb S. Zhao J. . (2023). Methylorubrum rhodesianum M520 as a biocontrol agent against Meloidogyne incognita (Tylenchida: Heteroderidae) J2s infecting cucumber roots. J. Appl. Microbiol. 134, lxad001. doi: 10.1093/jambio/lxad001 36611228

[B79] Zhou Q. Xiang Y. Li D. Luo X. Wu J. (2021). Global patterns and controls of soil nematode responses to nitrogen enrichment: a meta-analysis. Soil Biol. Biochem. 163, 108433. doi: 10.1016/j.soilbio.2021.108433 38826717

