# Supplementary material for: Cultivation type, season, and soil nematode interactions affect wheat rhizosphere metabarcoding profiles
Source: Front Plant Sci. 2026 Jul 16;17:1869384. doi: 10.3389/fpls.2026.1869384 (PMC13422436; doi:10.3389/fpls.2026.1869384)

Supplementary Figure 1 - Principal Component Analysis based on the correlation matrix of the soil physio-chemical variables per sample (see Supplementary Table 1 for details). First two components show samples aggregations in relation to the cultivation and sample types. Acronyms indicate samples from conventional (CONV) or organic (ORG) wheat fields, and related uncultivated controls (CONTR). The fraction of variance explained is shown along the axes. Analysis performed with PAST.

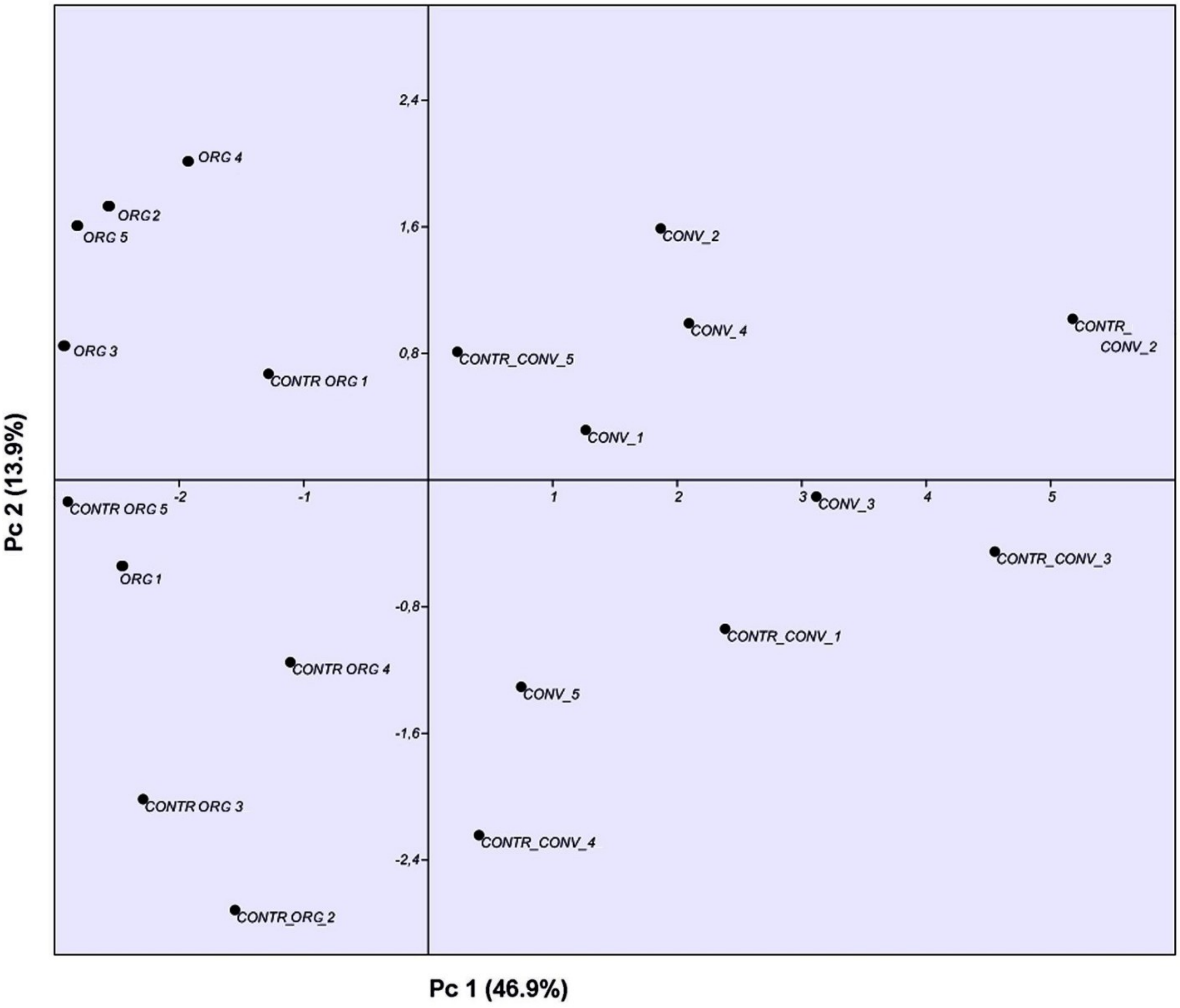

Supplement: Supplementary file 1 [file DataSheet1.pdf]
