# Supplementary material for: Cultivation type, season, and soil nematode interactions affect wheat rhizosphere metabarcoding profiles
Source: Front Plant Sci. 2026 Jul 16;17:1869384. doi: 10.3389/fpls.2026.1869384 (PMC13422436; doi:10.3389/fpls.2026.1869384)

Supplementary Figure 2 | Venn diagrams showing abundance and repartition of the ASV taxa in the sample groups from the conventional ("CONV.") and organic ("ORG.") wheat fields, including the background uncultivated controls from each field. **(A)** Plots of each sample group compared with the adjacent background controls at both samplings. **(B)** Diagrams showing the abundance variation in time for each group and **(C)** for all samples. Values show the number of taxa present in five replications samples per group after dataset filtering (1202 total ASV). Venn diagrams were produced on line at *http://bioinformatics.psb.ugent.be/webtools/Venn/* .

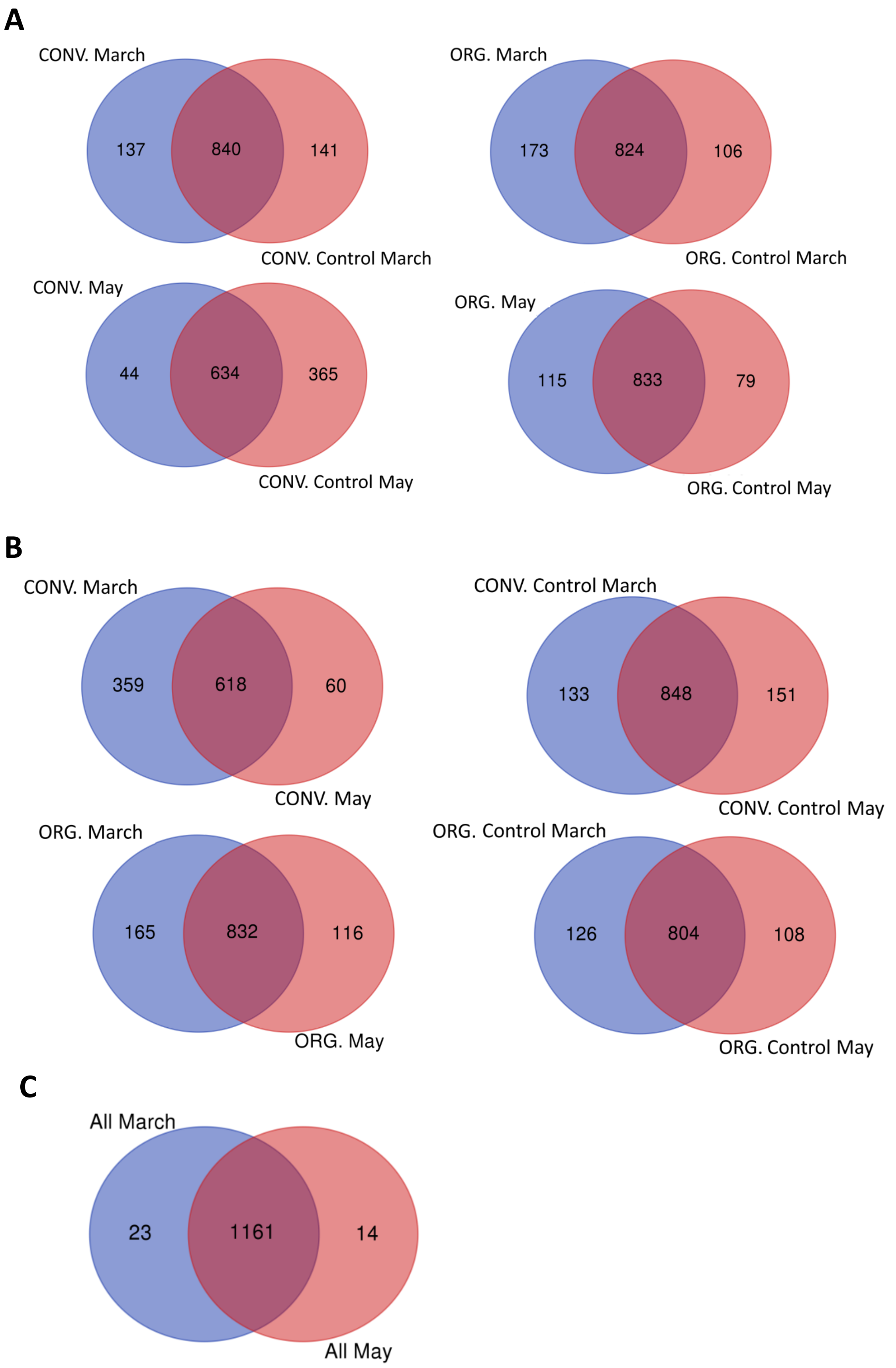

Supplement: Supplementary file 2 [file DataSheet2.pdf]
