# Supplementary material for: Cultivation type, season, and soil nematode interactions affect wheat rhizosphere metabarcoding profiles
Source: Front Plant Sci. 2026 Jul 16;17:1869384. doi: 10.3389/fpls.2026.1869384 (PMC13422436; doi:10.3389/fpls.2026.1869384)

Supplementary Figure 3 - NMDS plots showing sample groups homogeneity and partial overlapping of clusters, for most variables examined.

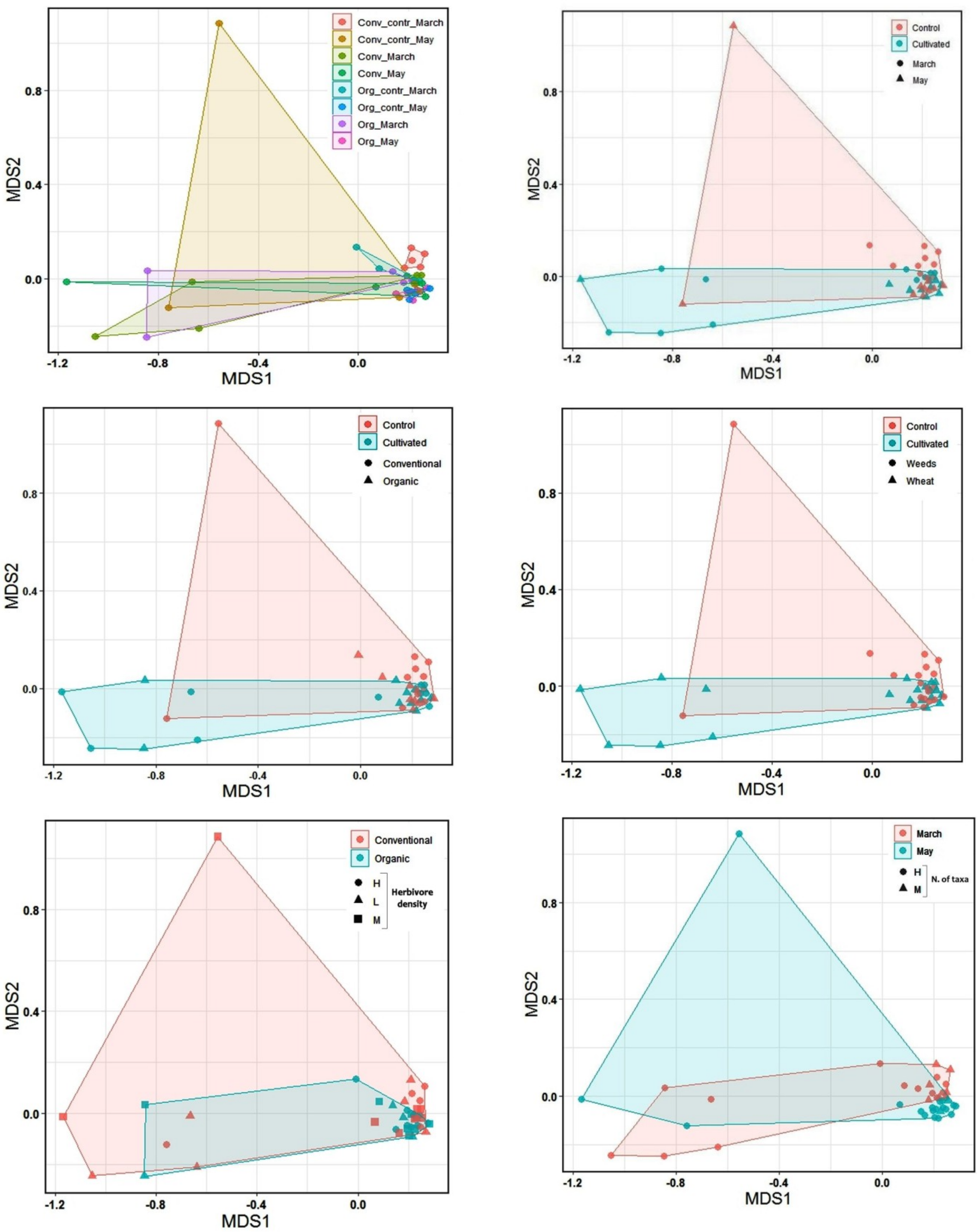

Supplement: Supplementary file 3 [file DataSheet3.pdf]
