# Supplementary material for: Cultivation type, season, and soil nematode interactions affect wheat rhizosphere metabarcoding profiles
Source: Front Plant Sci. 2026 Jul 16;17:1869384. doi: 10.3389/fpls.2026.1869384 (PMC13422436; doi:10.3389/fpls.2026.1869384)
Supplement: Supplementary file 4 [file DataSheet4.pdf]

Supplementary Figure 4 | Multivariate analyses of samples. Principal component analysis (PCA) based on the correlation matrix, and Cluster analysis (paired groups, Euclidean distance) based on the March (A, B) and May (C, D) ASV sequence abundance (filtered at 10000 total sequences per taxon). Variance explained by PC1 and PC2 = 46.5 and 10.5% (March), 45.6 and 10.2% (May). Analyses performed with PAST.

A)

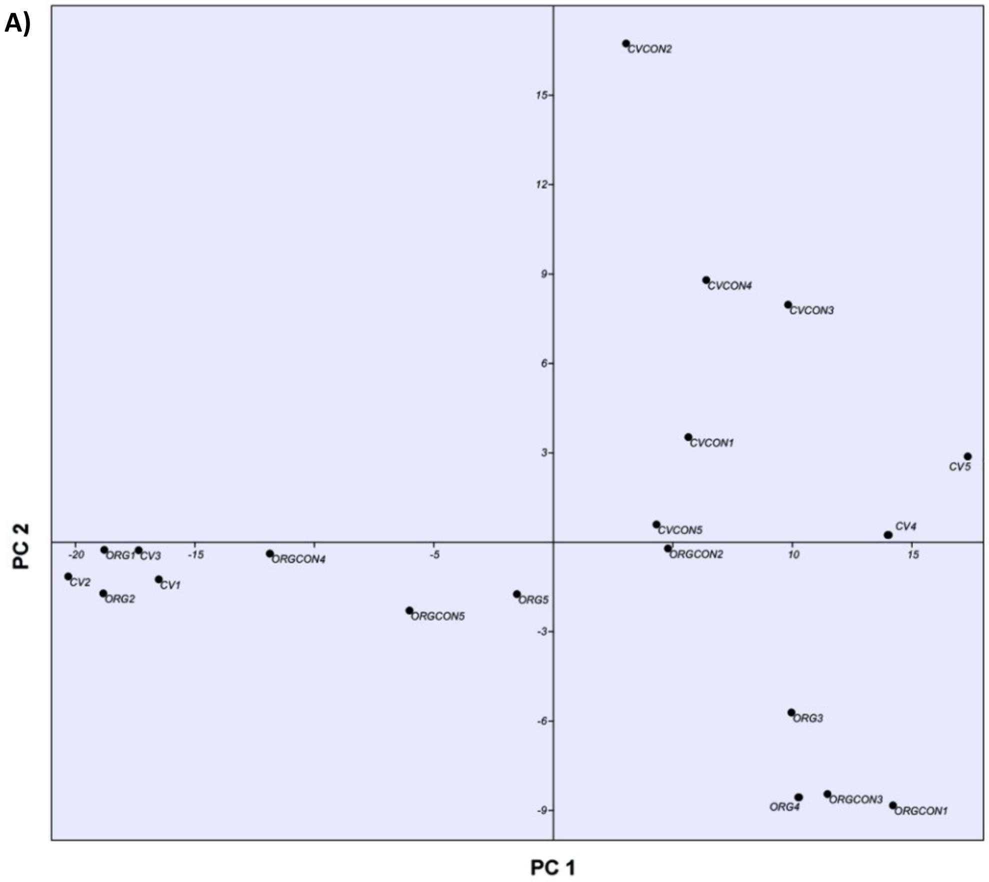

B)

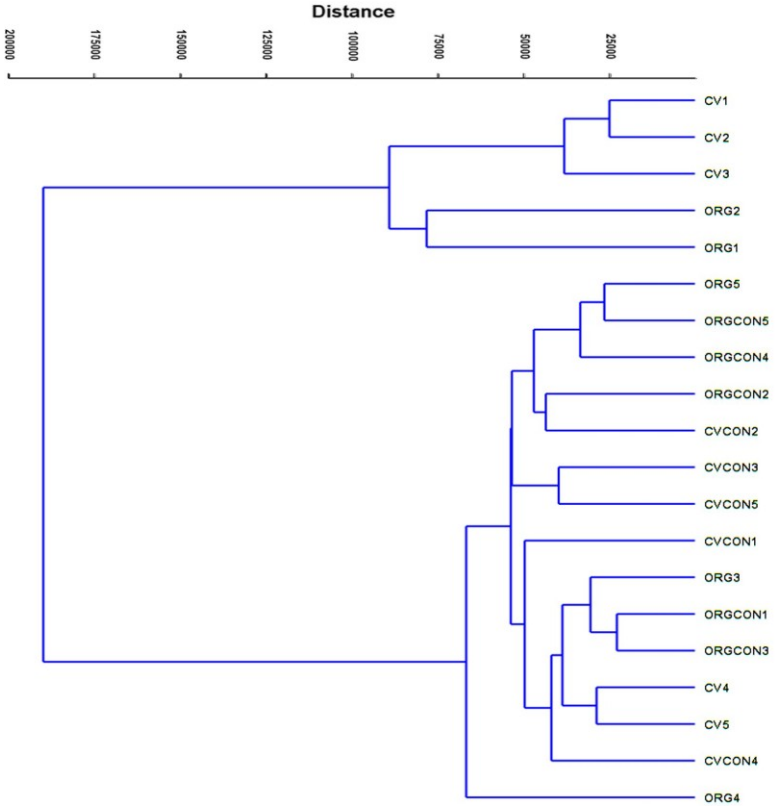

C)

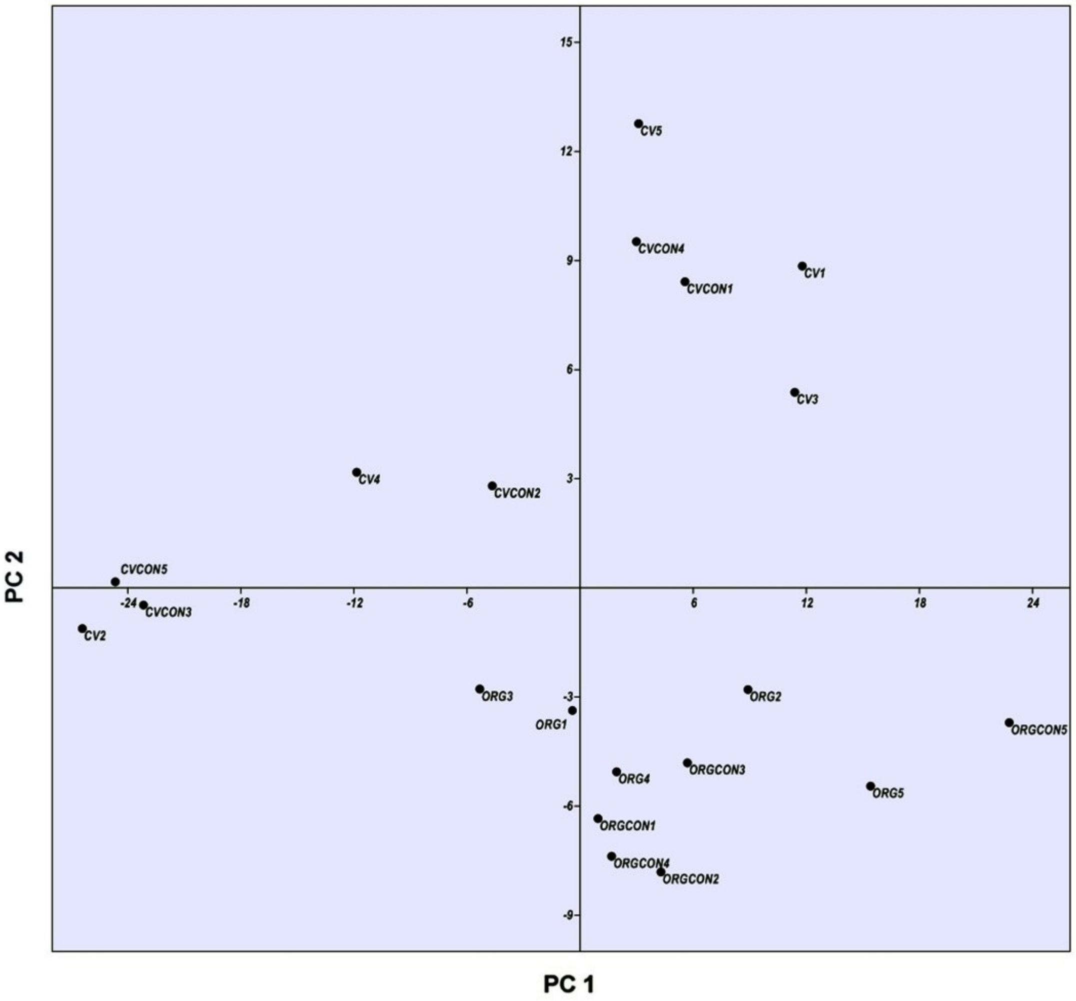

PC 2
PC 1
15
12
9
6
3
-3
-6
-9
-24
-18
-12
-6
6
12
18
24
CV5
CVCON4
CVCON1
CV1
CV3
CV4
CVCON2
CVCON5
CVCON3
CV2
ORG3
ORG1
ORG2
ORGCON5
ORG4
ORGCON3
ORG5
ORGCON1
ORGCON4
ORGCON2

D)

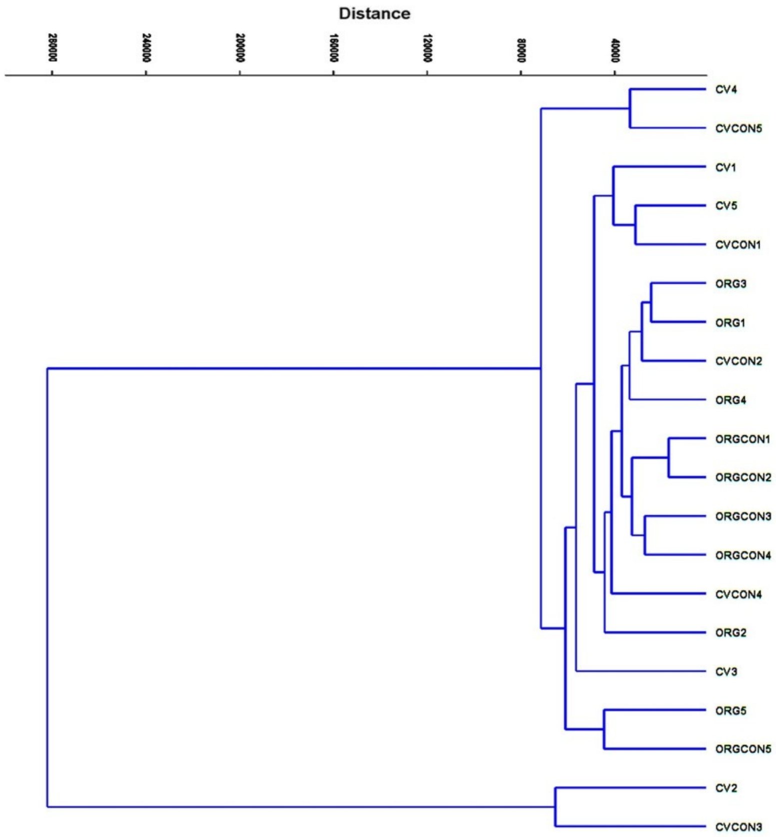

Distance
280000
240000
200000
160000
120000
80000
40000
CV4
CVCON5
CV1
CV5
CVCON1
ORG3
ORG1
CVCON2
ORG4
ORGCON1
ORGCON2
ORGCON3
ORGCON4
CVCON4
ORG2
CV3
ORG5
ORGCON5
CV2
CVCON3
