# Supplementary material for: Cultivation type, season, and soil nematode interactions affect wheat rhizosphere metabarcoding profiles
Source: Front Plant Sci. 2026 Jul 16;17:1869384. doi: 10.3389/fpls.2026.1869384 (PMC13422436; doi:10.3389/fpls.2026.1869384)
Supplement: Supplementary file 5 [file DataSheet5.pdf]

Supplementary Figure 5 - Seasonal effects observed on ASV metabarcoding profiles at the genus level, for the organic (A) and conventional (B) wheat samples.

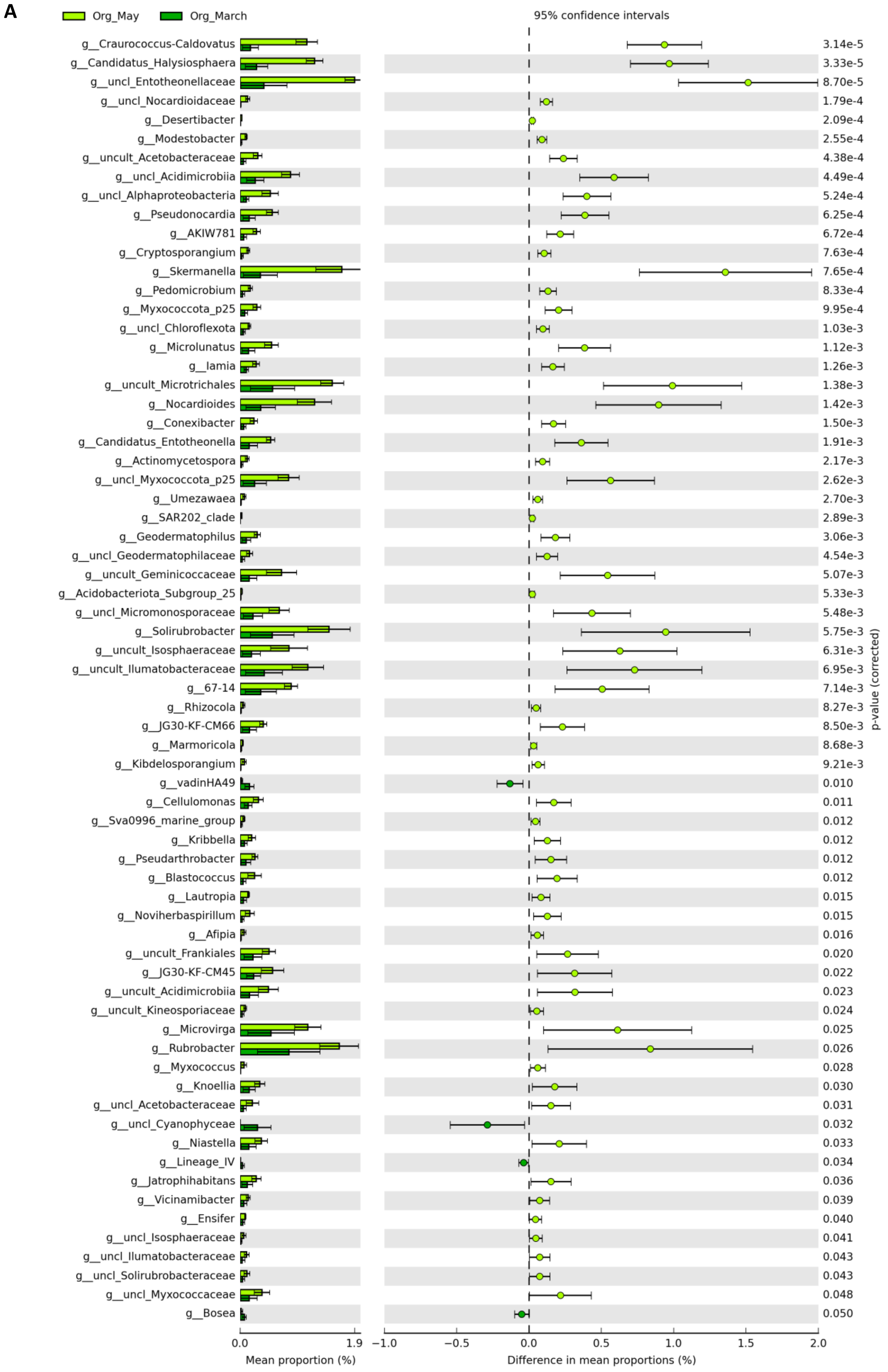

B

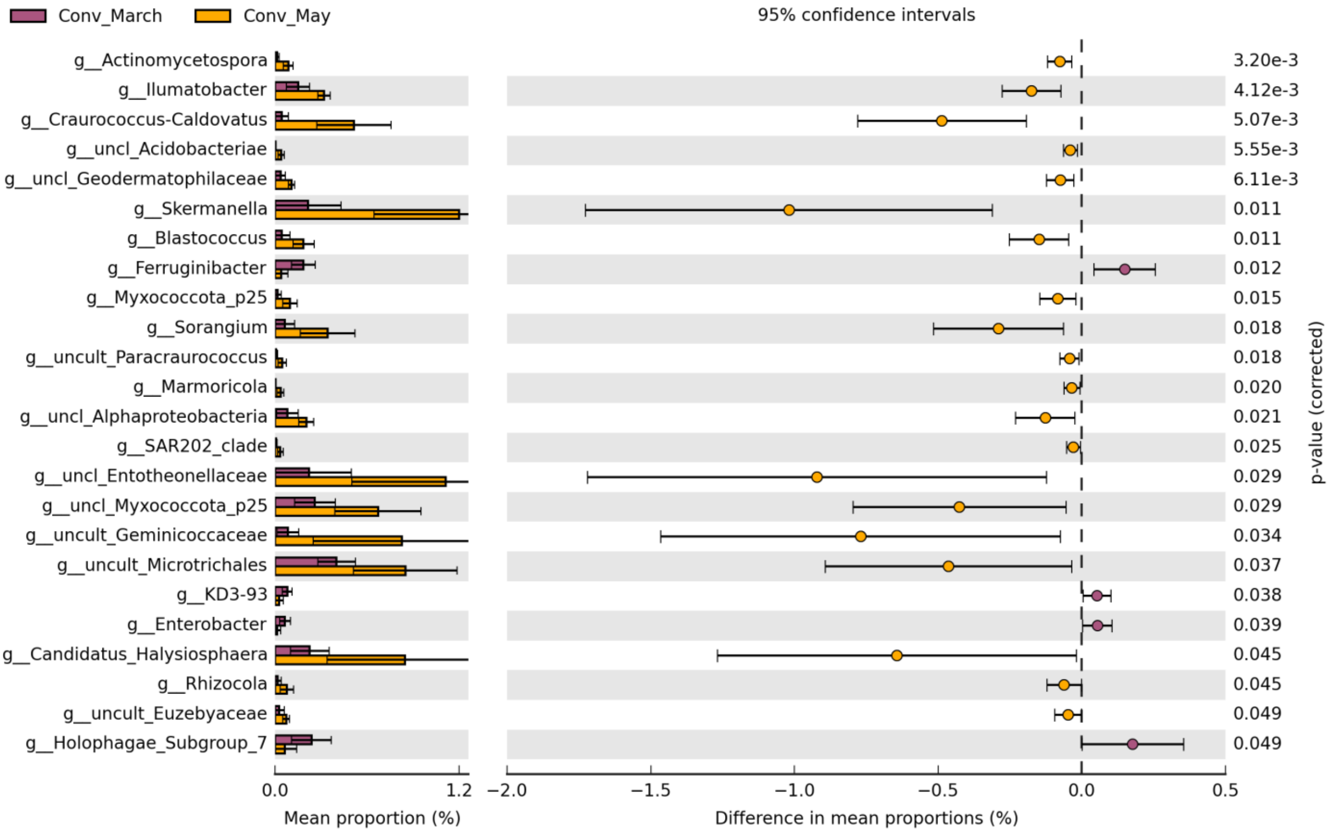
Conv_March
Conv_May
95% confidence intervals
g__Actinomycetospora
g__Ilumatobacter
g__Craurococcus-Caldovatus
g__uncl_Acidobacteriae
g__uncl_Geodermatophilaceae
g__Skermanella
g__Blastococcus
g__Ferruginibacter
g__Myxococcota_p25
g__Sorangium
g__uncult_Paracraurococcus
g__Marmoricola
g__uncl_Alphaproteobacteria
g__SAR202_clade
g__uncl_Entotheonellaceae
g__uncl_Myxococcota_p25
g__uncult_Geminicoccaceae
g__uncult_Microtrichales
g__KD3-93
g__Enterobacter
g__Candidatus_Halysiosphaera
g__Rhizocola
g__uncult_Euzebyaceae
g__Holophagae_Subgroup_7
3.20e-3
4.12e-3
5.07e-3
5.55e-3
6.11e-3
0.011
0.011
0.012
0.015
0.018
0.018
0.020
0.021
0.025
0.029
0.029
0.034
0.037
0.038
0.039
0.045
0.045
0.049
0.049
p-value (corrected)
0.0
1.2
Mean proportion (%)
−2.0
−1.5
−1.0
−0.5
0.0
0.5
Difference in mean proportions (%)
