# Supplementary material for: Cultivation type, season, and soil nematode interactions affect wheat rhizosphere metabarcoding profiles
Source: Front Plant Sci. 2026 Jul 16;17:1869384. doi: 10.3389/fpls.2026.1869384 (PMC13422436; doi:10.3389/fpls.2026.1869384)

**Supplementary Figure 6 -** ASV abundance as percent mean proportions, at the genus level, for samples classified high (H) or low (L) prevalence of fungal feeder nematodes in the whole nematode community.

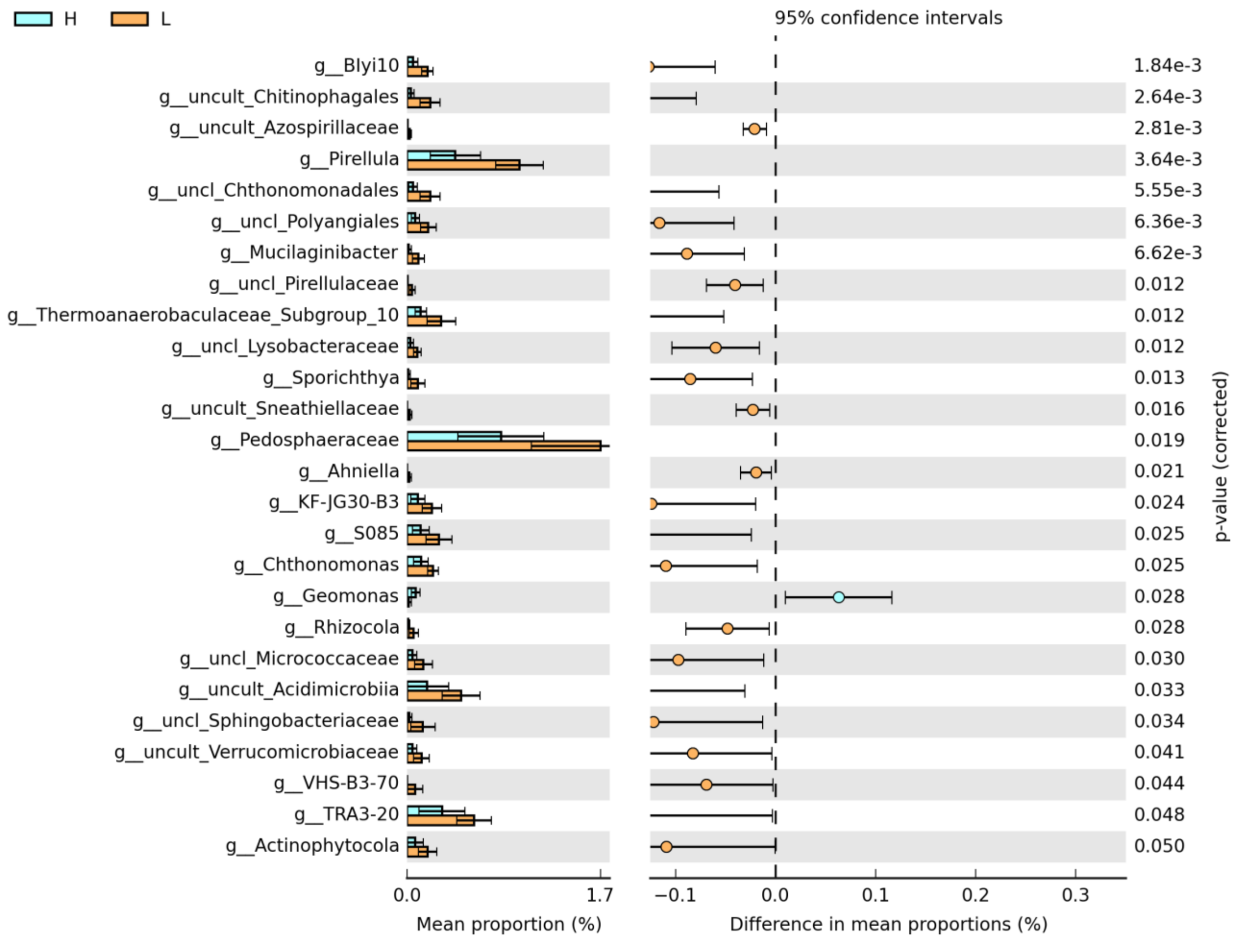

Supplement: Supplementary file 6 [file DataSheet6.pdf]
