# Supplementary material for: Cultivation type, season, and soil nematode interactions affect wheat rhizosphere metabarcoding profiles
Source: Front Plant Sci. 2026 Jul 16;17:1869384. doi: 10.3389/fpls.2026.1869384 (PMC13422436; doi:10.3389/fpls.2026.1869384)
Supplement: Supplementary file 7 [file DataSheet7.pdf]

Supplementary Figure 7 - Comparison of ASV abundance (percent mean proportions), at the order level, for samples from the organic (A) and conventional (B) wheat cultivations *vs* their corresponding controls, at both sampling time.

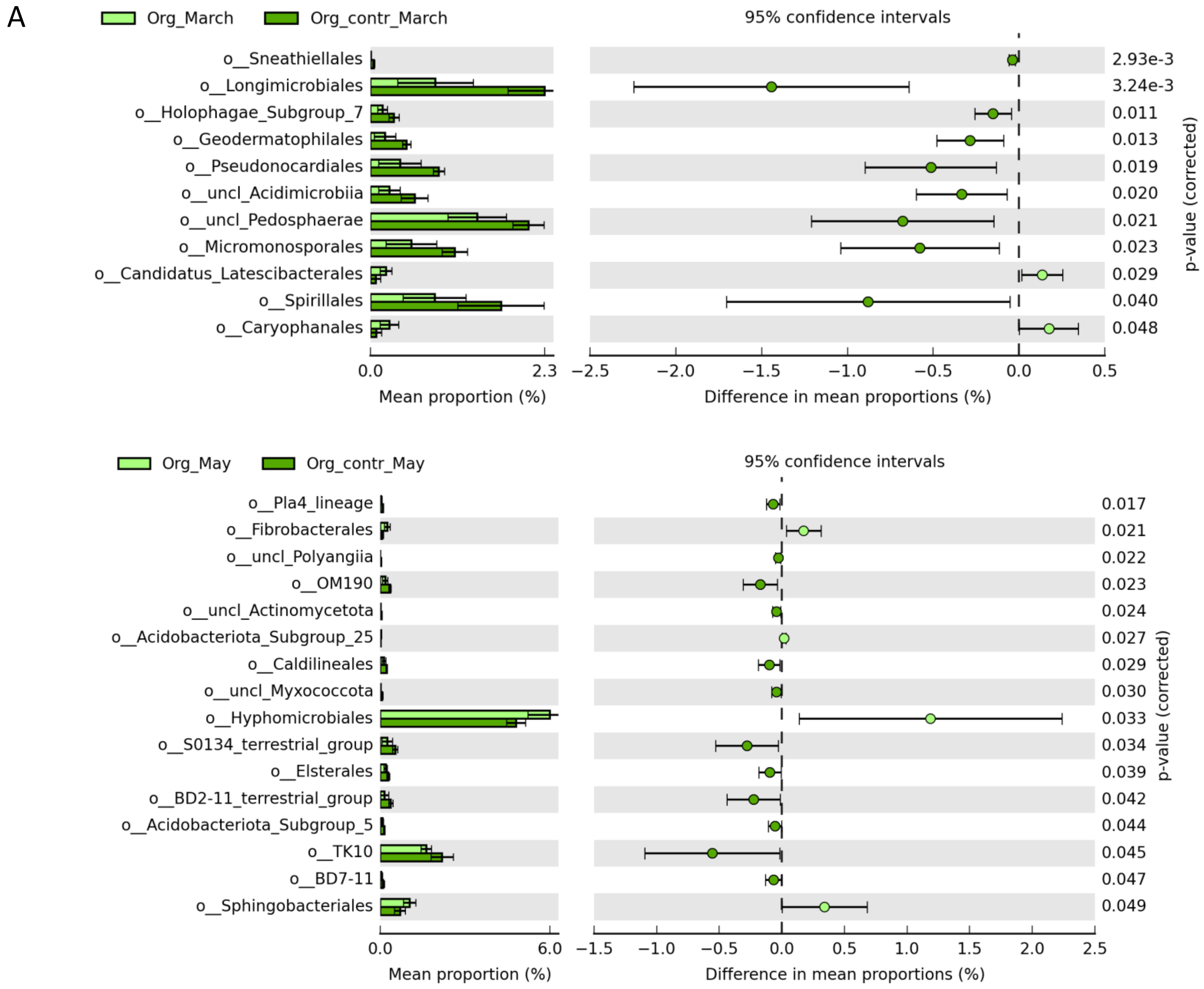

B

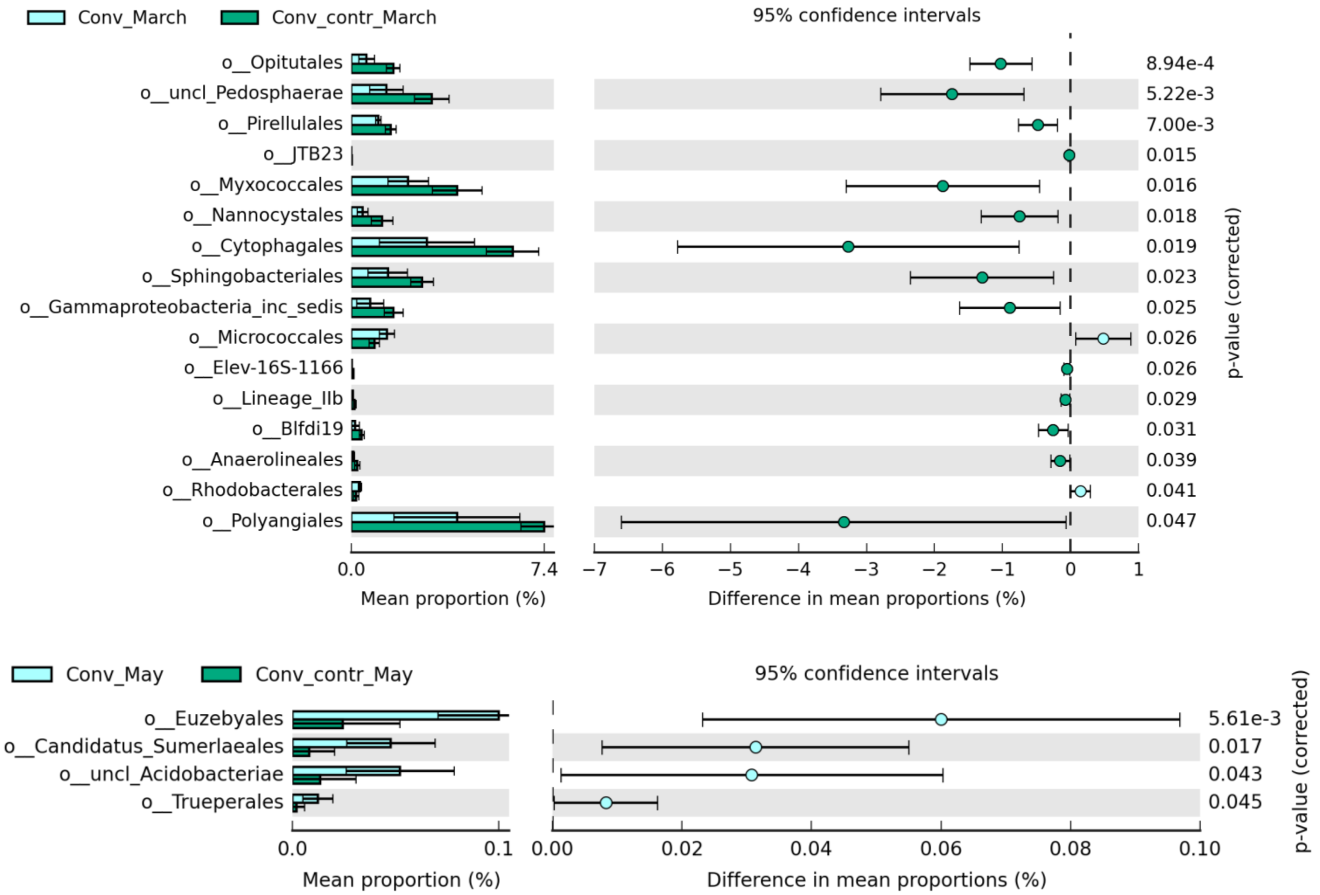
Conv_March
Conv_contr_March
95% confidence intervals
o__Opitutales
o__uncl_Pedosphaerae
o__Pirellulales
o__JTB23
o__Myxococcales
o__Nannocystales
o__Cytophagales
o__Sphingobacteriales
o__Gammaproteobacteria_inc_sedis
o__Micrococcales
o__Elev-16S-1166
o__Lineage_IIb
o__Blfdi19
o__Anaerolineales
o__Rhodobacterales
o__Polyangiales
8.94e-4
5.22e-3
7.00e-3
0.015
0.016
0.018
0.019
0.023
0.025
0.026
0.026
0.029
0.031
0.039
0.041
0.047
p-value (corrected)
0.0
7.4
Mean proportion (%)
−7
−6
−5
−4
−3
−2
−1
0
1
Difference in mean proportions (%)
Conv_May
Conv_contr_May
95% confidence intervals
o__Euzebyales
o__Candidatus_Sumerlaeales
o__uncl_Acidobacteriae
o__Trueperales
5.61e-3
0.017
0.043
0.045
p-value (corrected)
0.0
0.1
Mean proportion (%)
0.00
0.02
0.04
0.06
0.08
0.10
Difference in mean proportions (%)
