# Supplementary material for: Cultivation type, season, and soil nematode interactions affect wheat rhizosphere metabarcoding profiles
Source: Front Plant Sci. 2026 Jul 16;17:1869384. doi: 10.3389/fpls.2026.1869384 (PMC13422436; doi:10.3389/fpls.2026.1869384)
Supplement: Supplementary file 8 [file Table1.docx]

**Supplementary Table 4 -** Results of PERMANOVA pairwise comparisons of 1202 ASV sequence profiles in samples grouped by different classification variables (n. of samples = 40, based on Bray-Curtis dissimilarity matrix, calculated with R libraries *mctoolsr* and *vegan*).

| **Var1** | **Var2** | R^2^ | P ^a^ | P Bonferroni ^a^ | P FDR ^a^ |
| --- | --- | --- | --- | --- | --- |
| ***Cropping*** |  |  |  |  |  |
| Cultivated | Control | 0.05898 | 0.104 | 0.104 | 0.091 |
|  |  |  |  |  |  |
| ***Description*** |  |  |  |  |  |
| Organic | Conventional | 0.05898 | **0.045** | **0.045** | **0.04** |
|  |  |  |  |  |  |
| ***Plant*** |  |  |  |  |  |
| Weeds | Wheat | 0.0434 | 0.10 | 0.10 | 0.104 |
|  |  |  |  |  |  |
| ***Fert*** |  |  |  |  |  |
| Fertilized | Not fertilized | 0.05162 | 0.06 | 0.06 | 0.052 |
|  |  |  |  |  |  |
| ***Sampling time*** |  |  |  |  |  |
| March | May | 0.06721 | **0.018** | **0.018** | **0.022** |
|  |  |  |  |  |  |
| ***Herbivores, density* ^b^** |  |  |  |  |  |
| H | L | 0.08552 | 0.052 | 0.156 | 0.201 |
| H | M | 0.03210 | 0.462 | 0.467 | 0.467 |
| L | M | 0.04202 | 0.310 | 0.930 | 0.467 |
|  |  |  |  |  |  |
| ***Herbivores (%)* ^c^** |  |  |  |  |  |
| H | L | 0.07312382 | 0.094 | 0.282 | 0.312 |
| H | M | 0.05308155 | 0.319 | 0.957 | 0.366 |
| L | M | 0.03072578 | 0.347 | 1.000 | 0.366 |
|  |  |  |  |  |  |
| ***Fungal / moss feeders, density* ^b^** |  |  |  |  |  |
| H | L | 0.02554 | 0.589 | 1.000 | 0.74 |
| H | M | 0.02467 | 0.414 | 1.000 | 0.74 |
| L | M | 0.05042 | 0.715 | 1.000 | 0.74 |
|  |  |  |  |  |  |
| ***Fungal /moss feeders (%)* ^c^** |  |  |  |  |  |
| H | L | 0.17512304 | **0.042** | 0.126 | 0.117 |
| H | M | 0.03191619 | 0.361 | 1.000 | 0.353 |
| L | M | 0.04059571 | 0.162 | 0.486 | 0.216 |
|  |  |  |  |  |  |
| ***Bacterivores, density* ^b^** |  |  |  |  |  |
| H | L | 0.01326 | 1.000 | 1.000 | 1.000 |
| H | M | 0.01751 | 0.731 | 1.000 | 1.000 |
| L | M | 0.03787 | 1.000 | 1.000 | 1.000 |
|  |  |  |  |  |  |
| ***Bacterivores (%)* ^c^** |  |  |  |  |  |
| H | L | 0.04843162 | 0.388 | 1.000 | 0.532 |
| H | M | 0.03229554 | 0.549 | 1.000 | 0.532 |
| L | M | 0.03020285 | 0.285 | 0.855 | 0.532 |
|  |  |  |  |  |  |
| ***Omnivores-predators, density* ^b^** |  |  |  |  |  |
| H | L | 0.04414 | 0.247 | 0.741 | 0.3255 |
| H | M | 0.02263 | 0.568 | 1.000 | 0.5830 |
| L | M | 0.08861 | 0.104 | 0.312 | 0.3255 |
|  |  |  |  |  |  |
| ***Omnivores-predatory (%) ^c^*** |  |  |  |  |  |
| L | M | 0.04282792 | 0.1 | 0.1 | 0.115 |
|  |  |  |  |  |  |
| ***Number of nematode taxa*** |  |  |  |  |  |
| H | M | 0.0439263 | 0.07 | 0.07 | 0.083 |
|  |  |  |  |  |  |
| ***Soil type*** |  |  |  |  |  |
| clay | clay_loam | 0.02425166 | 0.39 | 0.39 | 0.382 |
|  |  |  |  |  |  |
| ***Description by time*** |  |  |  |  |  |
| Conventional, control, March | Conventional, control, May | 0.178656 | **0.01800** | 0.5040 | 0.06031 |
| Conventional, control, March | Conventional, wheat, March | 0.225415 | 0.17300 | 1.0000 | 0.22800 |
| Conventional, control, March | Conventional, wheat, May | 0.177066 | **0.01000** | 0.2800 | 0.05133 |
| Conventional, control, March | Organic, control, March | 0.172708 | **0.03500** | 0.9800 | 0.09000 |
| Conventional, control, March | Organic, control, May | 0.385796 | **0.01300** | 0.3640 | 0.05133 |
| Conventional, control, March | Organic, wheat, March | 0.198884 | **0.01300** | 0.3640 | 0.05133 |
| Conventional, control, March | Organic, wheat, May | 0.36833 | **0.00900** | 0.2520 | 0.05200 |
| Conventional, control, May | Conventional, wheat, March | 0.098226 | 0.36500 | 1.0000 | 0.42974 |
| Conventional, control, May | Conventional, wheat, May | 0.054965 | 0.85800 | 1.0000 | 0.87800 |
| Conventional, control, May | Organic, control, March | 0.150994 | 0.13600 | 1.0000 | 0.21778 |
| Conventional, control, May | Organic, control, May | 0.202458 | **0.00800** | 0.2240 | 0.05320 |
| Conventional, control, May | Organic, wheat, March | 0.088373 | 0.57300 | 1.0000 | 0.62462 |
| Conventional, control, May | Organic, wheat, May | 0.178481 | **0.05000** | 1.0000 | 0.09520 |
| Conventional, wheat, March | Conventional, wheat, May | 0.103215 | 0.40200 | 1.0000 | 0.49233 |
| Conventional, wheat, March | Organic, control, March | 0.209681 | 0.11400 | 1.0000 | 0.19775 |
| Conventional, wheat, March | Organic, control, May | 0.316007 | **0.00600** | 0.1680 | 0.05133 |
| Conventional, wheat, March | Organic, wheat, March | 0.062976 | 0.81600 | 1.0000 | 0.87111 |
| Conventional, wheat, March | Organic, wheat, May | 0.298222 | **0.01900** | 0.5320 | 0.06031 |
| Conventional, wheat, May | Organic, control, March | 0.140721 | 0.18000 | 1.0000 | 0.22800 |
| Conventional, wheat, May | Organic, control, May | 0.178184 | **0.01200** | 0.3360 | 0.05320 |
| Conventional, wheat, May | Organic, wheat, March | 0.087783 | 0.48000 | 1.0000 | 0.52528 |
| Conventional, wheat, May | Organic, wheat, May | 0.141749 | 0.14200 | 1.0000 | 0.21811 |
| Organic, control, March | Organic, control, May | 0.250001 | **0.01200** | 0.3360 | 0.05133 |
| Organic, control, March | Organic, wheat, March | 0.154606 | 0.21800 | 1.0000 | 0.30036 |
| Organic, control, March | Organic, wheat, May | 0.226406 | **0.01900** | 0.5320 | 0.05855 |
| Organic, control, May | Organic, wheat, March | 0.256785 | **0.00800** | 0.2240 | 0.05133 |
| Organic, control, May | Organic, wheat, May | 0.150723 | 0.14000 | 1.0000 | 0.21778 |
| Organic, wheat, March | Organic, wheat, May | 0.234106 | **0.01700** | 0.4760 | 0.05320 |

^a^ Significant values (P < 0.05) are shown in bold on grey background.

^b^  Nematodes in 100 ml soil, all samples, classified as L = low density (< 25 % of mean), M = medium density (25-75 % of mean), H = high density (> 75% of mean). All samples mean ± SD (min-max): Herbivores = March, 222 ± 279 (0-1040); May, 568 ± 731 (50-3250); Fungal / moss feeders = March, 257 ± 159 (33-633); May, 406 ± 222 (20-833); Bacterivores = March, 188 ± 99 (0-433); May, 265 ± 115 (75-520); Omnivores-predatory = March, 101 ± 108 (0-400); May, 292 ± 198 (20-715).

^c^ Group prevalence (%) in the whole nematode population. L = < 20%, M = 20-50%, H = > 50%.
