# Supplementary material for: Cultivation type, season, and soil nematode interactions affect wheat rhizosphere metabarcoding profiles
Source: Front Plant Sci. 2026 Jul 16;17:1869384. doi: 10.3389/fpls.2026.1869384 (PMC13422436; doi:10.3389/fpls.2026.1869384)
Supplement: Supplementary file 9 [file Table2.docx]

**Supplementary Table 5.** Taxa summary for more represented ASV at different levels, comparing samples grouped by classification variables. Significant *p* values (Kruskal-Wallis t-test, *p* < 0.05) for sample groups comparison (including Bonferroni and False Discovery Rate corrections) are shown in bold, with highest representations per groups shown in red (%). Dataset included a total of 1202 ASV (analyzed with R, library *mctoolsr*)*.*

| **Taxa** | *p* | *p* (Bonferroni) | *P* (FDR) | Variable: **Crop** | | | | | | | | | |  |  |  |
| --- | --- | --- | --- | --- | --- | --- | --- | --- | --- | --- | --- | --- | --- | --- | --- | --- |
|  |  |  |  | *Control* | | | | | *Cultivated* | | | | |  |  |  |
| c_Alphaproteobacteria | **0.006293987** | **0.03776392** | **0.03776392** | 0.1467 | | | | | 0.2777 | | | | |  |  |  |
| c_Deltaproteobacteria | **0.026547517** | 0.15928510 | 0.07964255 | 0.1003 | | | | | 0.0762 | | | | |  |  |  |
| o_Hyphomicrobiales | **0.002677193** | **0.01070877** | **0.01070877** | 0.0638 | | | | | 0.1553 | | | | |  |  |  |
| o_Sphingomonadales | **0.026547517** | 0.10619007 | 0.05309503 | 0.0289 | | | | | 0.0720 | | | | |  |  |  |
| o_Polyangiales | 0.194148451 | 0.77659381 | 0.25886460 | 0.0559 | | | | | 0.0456 | | | | |  |  |  |
| f_Methylobacteriaceae | **0.01282442** | **0.02564884** | **0.02564884** | 0.0316 | | | | | 0.1204 | | | | |  |  |  |
| f_Sphingomonadaceae | **0.01860473** | **0.03720945** | **0.01860473** | 0.0255 | | | | | 0.0695 | | | | |  |  |  |
| g_Sphingomonas | **0.01282442** | **0.02564884** | **0.02564884** | 0.0166 | | | | | 0.0634 | | | | |  |  |  |
|  |  |  |  | Variable: **Crop Description** | | | | | | | | | |  |  |  |
|  |  |  |  | *Conventional* | | | | | *Organic* | | | | |  |  |  |
| c__Gammaproteobacteria | **0.004507687** | **0.02253843** | **0.02253843** | 0.0580 | | | | | 0.0466 | | | | |  |  |  |
|  |  |  |  | Variable: **Plant type** | | | | | | | | | |  |  |  |
|  |  |  |  | *Weeds* | | | | | *Wheat* | | | | |  |  |  |
| c__Alphaproteobacteria | **0.00629398** | **0.0377639** | **0.0377639** | 0.1467 | | | | | 0.2777 | | | | |  |  |  |
| c__Deltaproteobacteria | **0.02654751** | 0.1592851 | 0.0796425 | 0.1003 | | | | | 0.0762 | | | | |  |  |  |
| o_Hyphomicrobiales | **0.00267719** | **0.0107087** | **0.0107087** | 0.0638 | | | | | 0.1553 | | | | |  |  |  |
| o_Sphingomonadales | **0.02654751** | 0.1061900 | 0.0530950 | 0.0289 | | | | | 0.0720 | | | | |  |  |  |
| f__Methylobacteriaceae | **0.0128244** | **0.0256488** | **0.0256488** | 0.0316 | | | | | 0.1204 | | | | |  |  |  |
| f__Sphingomonadaceae | **0.0186047** | **0.0372094** | **0.0186047** | 0.0255 | | | | | 0.0695 | | | | |  |  |  |
|  |  |  |  |  | | | | |  | | | | |  |  |  |
| g__Sphingomonas | **0.0128244** | **0.0256488** | **0.0256488** | 0.0166 | | | | | 0.0634 | | | | |  |  |  |
|  |  |  |  |  | | | | | | | | | |  |  |  |
|  |  |  |  |  | | | | | | | | | |  |  |  |
|  |  |  |  | Variable: **Sampling time** | | | | | | | | | |  |  |  |
|  |  |  |  | *March* | | | | *May* | | | | | |  |  |  |
| c__Actinomycetes | **2.167835e-05** | **0.0001083** | **0.0001083** | 0.0418 | | | | 0.0788 | | | | | |  |  |  |
| c__Betaproteobacteria | **2.448697e-03** | **0.01224348** | **0.0061217** | 0.0562 | | | | 0.0430 | | | | | |  |  |  |
| c__Gammaproteobacteria | **5.333667e-03** | **0.02666833** | **0.0088894** | 0.0580 | | | | 0.0466 | | | | | |  |  |  |
| o_Rhodospirillales | **4.843261e-06** | **1.937304e-05** | **1.9373e-05** | 0.0171 | | | | 0.0543 | | | | | |  |  |  |
| o_Sphingomonadales | **7.407233e-03** | **2.962893e-02** | **1.4814e-02** | 0.0626 | | | | 0.0383 | | | | | |  |  |  |
| o_Polyangiales | 5.146208e-02 | 2.058483e-01 | 6.8616e-02 | 0.0558 | | | | 0.0457 | | | | | |  |  |  |
| o_Hyphomicrobiales | 5.162077e-01 | 2.064831e+00 | 5.1620e-01 | 0.1236 | | | | 0.0955 | | | | | |  |  |  |
| f_Sphingomonadaceae | **0.00579591** | **0.01159184** | **0.0115918** | 0.0598 | | | | 0.0352 | | | | | |  |  |  |
| g_Sphingomonas | **0.01282442** | **0.02564884** | **0.0256488** | 0.0525 | | | | 0.0275 | | | | | |  |  |  |
|  |  |  |  | Variable: **Soil type** | | | | | | | | | |  |  |  |
|  |  |  |  | *Clay* | | | | | *Clay-loam* | | | | |  |  |  |
| c_Gammaproteobacteria | **0.01211778** | 0.06058892 | 0.06058892 | 0.0442 | | | | | 0.0558 | | | | |  |  |  |
| c_Betaproteobacteria | **0.01972115** | 0.09860575 | **0.04930288** | 0.0433 | | | | | 0.0523 | | | | |  |  |  |
| o_Hyphomicrobiales | **0.00662169** | **0.01986508** | **0.01986508** | 0.1090 | | | | | 0.1098 | | | | |  |  |  |
| f_Methylobacteriaceae | **0.03612668** | 0.07225336 | 0.07225336 | 0.0682 | | | | | 0.0794 | | | | |  |  |  |
|  |  |  |  | Variable: **Herbivores**^a^ | | | | | | | | | |  |  |  |
|  |  |  |  | *H* | | *L* | | | | | *M* | | |  |  |  |
| o_Sphingomonadales | **0.02133234** | 0.08532937 | 0.08532937 | 0.0284 | | 0.0699 | | | | | 0.0567 | | |  |  |  |
| f_Sphingomonadaceae | 0.02456152 | 0.07368456 | 0.07368456 | 0.02555872 | | 0.06728142 | | | | | 0.05369379 | | |  |  |  |
| g_Sphingomonas | **0.0101268** | **0.0202537** | **0.0202537** | 0.0188 | | 0.0611 | | | | | 0.0443 | | |  |  |  |
|  |  |  |  | Variable: **Herbivores (%)^b^** | | | | | | | | | |  |  |  |
|  |  |  |  | H | | L | | | | | M | | |  |  |  |
| c__Gammaproteobacteria | **0.0332353** | 0.1661769 | 0.1661769 | 0.0613 | | 0.0545 | | | | | 0.0455 | | |  |  |  |
| o_Hyphomicrobiales | **0.0247275** | 0.07418264 | 0.07418264 | 0.0381 | | 0.1385 | | | | | 0.1082 | | |  |  |  |
| f_Methylobacteriaceae | **0.0334864** | 0.0669729 | 0.06697290 | 0.00635 | | 0.1042 | | | | | 0.0748 | | |  |  |  |
| g_Sphingomonas | **0.0273637** | 0.05472748 | 0.05472748 | 0.00760 | | 0.0572 | | | | | 0.0345 | | |  |  |  |
| g_Methylobacterium-Methylorubrum | **0.0288804** | 0.05776088 | 0.02888044 | 0.0 | | 0.0927 | | | | | 0.0645 | | |  |  |  |
|  |  |  |  | Variable: **Bacterivores (%)^b^** | | | | | | | | | |  |  |  |
|  |  |  |  | *H* | | *L* | | | | | *M* | | |  |  |  |
| g_Methylobacterium-Methylorubrum | **0.0231202** | **0.0462405** | **0.0462405** | 0.0 | | 0.0450 | | | | | 0.0870 | | |  |  |  |
|  |  |  |  | Variable: **Fungivores (%)^b^** | | | | | | | | | |  |  |  |
|  |  |  |  | *H* | | *L* | | | | | *M* | | |  |  |  |
| c_Alphaproteobacteria | **0.0481140** | 0.2886843 | 0.2886843 | 0.2904 | | 0.1075 | | | | | 0.2244 | | |  |  |  |
| o_Hyphomicrobiales | **0.0139813** | 0.0559251 | 0.0559251 | 0.1690 | | 0.0373 | | | | | 0.1170 | | |  |  |  |
| f_Methylobacteriaceae | **0.0136003** | **0.0272007** | **0.0272007** | 0.1344 | | 0.0058 | | | | | 0.0831 | | |  |  |  |
|  |  |  |  | Variable: **Omnivores-predators (%)^b^** | | | | | | | | | |  |  |  |
|  |  |  |  | *H* | | *L* | | | | | *M* | | |  |  |  |
| c__Deltaproteobacteria | **0.0257951** | 0.1289758 | 0.1289758 | - | | 0.0985 | | | | | 0.0757 | | |  |  |  |
|  |  |  |  | Variable: **N. of nematode taxa per sample**^c^ | | | | | | | | | |  |  |  |
|  |  |  |  | *H* | | | | | *M* | | | | |  |  |  |
| c_Alphaproteobacteria | **0.00835482** | **0.0584837** | 0.05848379 | 0.2384 | | | | | 0.1074 | | | | |  |  |  |
| c_Deltaproteobacteria | **0.02563927** | 0.1794749 | 0.08973745 | 0.0824 | | | | | 0.1117 | | | | |  |  |  |
| c_Gammaproteobacteria | **0.03604832** | 0.2523382 | 0.08411276 | 0.0500 | | | | | 0.0616 | | | | |  |  |  |
| o_Hyphomicrobiales | **0.02563927** | 0.1025571 | 0.10255709 | 0.1262 | | | | | 0.0429 | | | | |  |  |  |
| o_Polyangiales | **0.03315556** | 0.1326222 | 0.06631112 | 0.0470 | | | | | 0.0656 | | | | |  |  |  |
| f_Methylobacteriaceae | **0.01491205** | **0.0298241** | **0.0298241** | 0.0923 | | | | | 0.0109 | | | | |  |  |  |
|  |  |  |  | Variable: **Description & sampling time** | | | | | | | | | | | | |
|  |  |  |  | Conv. cont. March | Conv. cont. May | | Conv. March | | | Conv. May | | Org. cont. March | Org. cont. May | | Org. March | Org. May |
| c_Thermoleophilia | **0.0018861** | **0.016975** | **0.0169752** | 0.0141 | 0.0302 | | 0.0204 | | | 0.0323 | | 0.0256 | 0.0501 | | 0.0229 | 0.0504 |
| c_Actinomycetes | **0.0021499** | **0.019349** | **0.0096747** | 0.0337 | 0.0685 | | 0.0390 | | | 0.0737 | | 0.0584 | 0.0853 | | 0.0362 | 0.0878 |
| c_Acidimicrobiia | **0.0022684** | **0.020415** | **0.0068052** | 0.0280 | 0.0320 | | 0.0219 | | | 0.0387 | | 0.0345 | 0.0470 | | 0.0226 | 0.0541 |
| c_Gammaproteobacteria | **0.0051632** | **0.046469** | **0.0116174** | 0.0692 | 0.0525 | | 0.0546 | | | 0.0557 | | 0.0593 | 0.0371 | | 0.0489 | 0.0413 |
| c_Alphaproteobacteria | **0.0109207** | 0.098286 | **0.0196573** | 0.0764 | 0.2556 | | 0.3544 | | | 0.2698 | | 0.1188 | 0.1362 | | 0.3298 | 0.1567 |
| c_Cytophagia | **0.0158145** | 0.142330 | **0.0237217** | 0.0614 | 0.0293 | | 0.0287 | | | 0.0374 | | 0.0577 | 0.0291 | | 0.0361 | 0.0356 |
| c_Betaproteobacteria | **0.0193075** | 0.173768 | **0.0248240** | 0.0662 | 0.0436 | | 0.0542 | | | 0.0452 | | 0.0590 | 0.0423 | | 0.0455 | 0.0409 |
| o_Rhodospirillales | **0.000912** | **0.00547** | **0.005** | 0.0157 | 0.0530 | | 0.0107 | | | 0.0476 | | 0.0259 | 0.0542 | | 0.0163 | 0.0624 |
| o_Hyphomicrobiales | **0.006558** | **0.03934** | **0.0196** | 0.0276 | 0.1326 | | 0.2385 | | | 0.1416 | | 0.0471 | 0.0480 | | 0.1812 | 0.0599 |
| o_Sphingomonadales | **0.014115** | 0.084695 | **0.028231** | 0.0180 | 0.0577 | | 0.0895 | | | 0.0639 | | 0.0256 | 0.0143 | | 0.1173 | 0.0173 |
| o_Cytophagales | **0.015814** | 0.0948871 | **0.02372** | 0.0614 | 0.0293 | | 0.0287 | | | 0.0374 | | 0.0577 | 0.0291 | | 0.0361 | 0.0356 |
| f_Sphingomonadaceae | **0.0070171** | 0.021051 | **0.021051** | 0.0154 | 0.0540 | | 0.0880 | | | 0.0619 | | 0.0215 | 0.0113 | | 0.1145 | 0.0138 |
| f_Methylobacteriaceae | **0.0123452** | **0.037035** | **0.018517** | 0.0024 | 0.1055 | | 0.2123 | | | 0.1096 | | 0.0083 | 0.0103 | | 0.1468 | 0.0128 |
| g_Sphingomonas | **0.01605756** | **0.048172** | **0.048172** | 0.0073 | 0.0408 | | 0.0817 | | | 0.0554 | | 0.0123 | 6.00e-03 | | 0.1086 | 8.08e-03 |
| g_Methylobacterium-Methylorubrum | **0.02709025** | 0.081270 | **0.040635** | 0.0000 | 0.0945 | | 0.1990 | | | 0.0987 | | 0.0004 | 6.94e-05 | | 0.1344 | 9.99e-05 |
|  |  |  |  |  |  | |  | | |  | |  |  | |  |  |

^a^ Nematodes in 100 ml soil, all samples, classified as L = low density (< 25 % of mean), M = medium density (25-75 % of mean), H = high density (> 75% of mean). All samples mean ± SD (min-max): Herbivores = March, 222 ± 279 (0-1040); May, 568 ± 731 (50-3250); Fungivores-moss feeders = March, 257 ± 159 (33-633); May, 406 ± 222 (20-833); Bacterivores = March, 188 ± 99 (0-433); May, 265 ± 115 (75-520); Omnivores-predatory = March, 101 ± 108 (0-400); May, 292 ± 198 (20-715).

^b^ Group prevalence (%) in the whole nematode population. L = < 20%, M = 20-50%, H = > 50%.

^c^  Mean = 12, High (H) = >
